# The legalization of international instruments: a hybrid RAG scoring framework based on chain-of-thought prompting

**DOI:** 10.3389/frai.2026.1784200

**Published:** 2026-07-09

**Authors:** Yan Chen, Zihua Zeng, Muhamad Sayuti Hassan

**Affiliations:** 1 Faculty of Law, Universiti Kebangsaan Malaysia, Bangi, Malaysia; 2 School of Computer Science, The University of Sydney, Sydney, NSW, Australia

**Keywords:** chain-of-thought prompting, international instruments, large language models, legalization, retrieval-augmented generation

## Abstract

**Introduction:**

Accurately evaluating the degree of legalization of international instruments is analytically valuable for assessing the level of institutionalization of interstate cooperative arrangements. However, conventional assessment approaches rely heavily on specialized legal expertise, and the inherent efficiency constraints of manual analysis make systematic evaluation across large-scale instrument corpora exceedingly difficult.

**Methods:**

To address the lack of automated scoring tools in this domain, this study introduces a hybrid retrieval-augmented generation (RAG) scoring framework based on chain-of-thought (CoT) prompting. Based on the legalization conceptual framework proposed by Abbott and Snidal, this study constructs a five-level coding standard that covers the three dimensions of obligation, precision, and delegation, and accordingly designs a stepwise binary decision process to develop the corresponding CoT prompt template. In parallel, this study constructs a vector-indexed clause database comprising 2,611 expert-annotated samples from the ASEAN instrument corpus, providing retrieval-augmented semantic references for the scoring task. The retrieval mechanism incorporates an adaptive quality-weighted ranking strategy and employs an additional pre-trained model to perform secondary filtering of candidate results. To evaluate the effectiveness of the scoring framework, two independent test sets were constructed from ASEAN instruments and documents from other major regional organizations, respectively, containing 254 and 255 expert-annotated samples.

**Results:**

GPT-5.2 and GPT-4o, based on this architecture, significantly outperform the baseline prompting strategy, traditional machine learning methods (TF-IDF+LR), and the fine-tuned pre-trained language model (Legal-BERT) on the international instrument scoring task. Among all evaluated models, GPT-5.2 exhibits the strongest overall performance. Based on the averaged outcomes of three independent runs, the model attained QWK scores of 0.806 and 0.788, and MAE scores of 0.089 and 0.084, on the ASEAN instrument test set and the test set of other major regional organizations, respectively, demonstrating a substantially high degree of agreement with human expert ratings.

**Discussion:**

These findings indicate that integrating CoT prompting with the RAG pipeline improves LLM performance in scoring the degree of legalization of international legal documents. This provides methodological and technical foundations for large-scale institutional empirical analysis in ASEAN and broader legal systems, and points toward future extensions of the framework to other regional and multilateral legal orders.

## 1 Introduction

Since the advent of legalization in international relations, the drafting and formulation of international instruments have attracted sustained scholarly attention for their critical roles in institutional formation, norm development, and the articulation of political intent (Goldstein et al., 2000). In particular, as transnational issues such as environmental protection, economic cooperation, and transnational crime have proliferated, states have increasingly sought to institutionalize cooperation through bilateral or multilateral instruments to address complex cross-border and regional challenges jointly. These instruments typically originate as declaratory political instruments embodying soft law characteristics, but tend to evolve into legal instruments that embody complex legal characteristics as cooperation among contracting parties deepens over time. However, due to sovereignty concerns or pragmatic political considerations, some states in certain regions have declined to establish formal institutions or mechanisms, opting instead for more flexible and informal forms of cooperation. Within this context, examining the legalization of international instruments offers insights not only into how varying degrees of legalization affect state compliance but also into the institutional logic underlying states' choices of particular legalization pathways across historical periods and issue domains (Bélanger and Fontaine-Skronski, 2012).

To render the concept of “legalization” analytically operational, Abbott et al. (2000). proposed an analytical framework encompassing three dimensions to measure the overall constraining characteristics of international arrangements. Within this framework, obligation refers to the extent to which commitments are legally binding; precision denotes the clarity and determinacy of rule content; and delegation concerns the conferral of interpretative, adjudicative, or implementation authority upon third-party bodies. By dichotomizing each of the three dimensions (high/low), the authors constructed eight theoretical types to illustrate the gradual, hierarchical nature of legalization within international arrangements more intuitively. Building upon this foundation, subsequent studies have further developed various derivative coding standards, which have been widely applied in empirical research across fields such as economics (Berge, 2020), environment (Böhmelt and Butkute, 2018), and political security (Kusumawardhana, 2022). These studies quantitatively analyzed international instruments concluded between states to reveal the characteristics and dynamic evolution of legalization within international arrangements. However, due to significant differences in coding standards across studies, such legalization coding practices suffer from notable limitations in cross-study comparability and reproducibility of results. Meanwhile, the manual coding methods employed in this research are not only susceptible to researchers' subjective biases but also constrained by high labor costs, limiting their applicability to large-scale sample analyses. Consequently, existing studies on international instruments often feature small sample sizes and limited data coverage, making it challenging to comprehensively capture the overall characteristics of legalization in international arrangements.

Recent advances in Natural Language Processing (NLP) have opened new avenues for research in this domain. In particular, recent advancements in Large Language Models (LLMs) have demonstrated remarkable potential in handling complex legal tasks, including case analysis, drafting legal documents, and reviewing contracts (Linardatos et al., 2020). The remarkable advances in LLMs' semantic understanding and text representation can reduce some sources of human inconsistency in manual coding and enhance coding efficiency and scalability. Nevertheless, with respect to the existing body of research, the application of LLMs to the scoring of international instruments remains at an early stage of methodological exploration, characterized by the absence of both established scoring frameworks and empirically validated quantitative assessment approaches. This limitation largely stems from the fact that current general-purpose LLMs exhibit insufficient domain-specific expertise in law and politics, limited semantic discrimination capabilities, and shallow causal reasoning depth, thereby constraining their ability to identify and quantitatively evaluate complex clauses within international instruments accurately (Shao et al., 2025). At the same time, the pervasive “hallucination” phenomenon in LLM-generated texts significantly undermines their reliability and practical applicability in high-precision, low-tolerance domains such as legal reasoning and analysis (Dahl et al., 2024). In addition, model outputs may be affected by differences in model versions and prompting designs, introducing variability and bias. In high-risk application scenarios such as law, this uncertainty may further bring multidimensional trust-related challenges, including issues of legality, ethics, and robustness (Siddharth and Luo, 2025).

To address these challenges, this study introduces a scoring framework that integrates the RAG pipeline with CoT prompting, thereby enhancing LLMs' knowledge accuracy and reasoning coherence in clause-level scoring tasks. Building on Abbott et al.'s influential “legalization” framework, this study develops a five-level coding standard encompassing the three dimensions of obligation, precision, and delegation, along with a stepwise binary decision process. The proposed coding standard and decision process provide standardized, operational guidelines for expert annotation, thereby ensuring the development of a high-quality vector-indexed clause database. Simultaneously, embedding them as CoT prompting templates within model inputs strengthens the accuracy and consistency of LLMs‘ outputs in clause-level scoring tasks. At the retrieval mechanism level, a novel “quality-weighted ranking” strategy is introduced that integrates semantic similarity with scoring confidence to dynamically reweight and optimize the ranking of candidate clause embeddings, thereby enhancing both relevance and epistemic reliability during the knowledge retrieval phase. Furthermore, a pretrained model is incorporated into the retrieval pipeline to conduct secondary filtering based on semantic coherence and confidence estimation, effectively eliminating noisy samples and weakly relevant instances. This study aims to construct a clause-level scoring framework for assessing the degree of legalization of international instruments by integrating a structured coding system, expert-annotated data, and a retrieval-augmented reasoning mechanism, to address the limitations of traditional manual coding standards being fragmented, scoring being highly subjective, and existing automated methods lacking stable reasoning structures. The specific contributions of this study can be summarized as follows:

(1) This study proposes a quantitative method based on a “five-level coding standard” and a “stepwise binary decision process,” and constructs a reusable and highly operational coding rule system for evaluating the degree of legalization of international instruments.

(2) This study constructs an expert-annotated benchmark dataset encompassing instruments from ASEAN and other major regional organizations, thereby furnishing a reproducible and verifiable empirical foundation for the systematic evaluation of scoring accuracy and inter-rater reliability among human experts and large language models in the context of international instrument scoring tasks.

(3) This study empirically demonstrates that a hybrid scoring framework integrating CoT prompting and a RAG pipeline outperforms baseline methods in international instrument scoring tasks, achieving near-expert inter-rater agreement on both in-domain and cross-domain test sets under the optimal model configuration.

In summary, the study constructs and empirically validates a scoring framework based on a standardized coding rule system and a high-quality expert-annotated benchmark dataset, which provides technical support for large-scale empirical analysis of legalization in international legal documents, and also offers methodological insights and an experiential foundation for the interdisciplinary application and expansion of LLMs in the legal domain.

## 2 Related work

### 2.1 Coding scheme for the legalization of international instruments

Developing a systematic coding scheme to assess the degree of legalization of international instruments constitutes a crucial analytical approach for examining the institutional logic and operational dynamics of international arrangements. In this area, the academic community has conducted extensive research and accumulated a wealth of findings. Overall, existing studies can be divided into two main approaches: qualitative research, which focuses on theoretical interpretation and case studies, and quantitative research, which emphasizes measurement and modeling analysis.

Qualitative research is typically based on the legalization analytical framework, which involves conducting meticulous textual interpretation and manual coding of legal provisions to achieve preliminary quantification of legalization levels. This approach thereby uncovers the institutional logic, normative orientation, and formative mechanisms embedded in international instruments. In their analysis of the ASEAN Convention Against Trafficking in Persons, Wirawan and Novikrizna coded its clauses along three dimensions, assigning values (high = 2, low = 1), and applied a weighted-aggregation method to compute the document's overall legalization index (Wirawan and Novikrisna, 2024). Muhammad adopted a comparable approach in his analysis of the ASEAN instrument on Transboundary Haze Pollution. Although explicit numerical coding was not applied, the author conducted a clause-by-clause qualitative assessment of the relative strength of obligation, precision, and delegation, thereby elucidating the main institutional design characteristics of the treaty (Muhammad, 2022). Furthermore, in the study of EU soft law, Terpan established a coding model for classifying legal norms based on an “obligation–delegation” binary framework, using the strength of normative binding force and enforcement mechanisms to delineate the boundary between soft and hard law (Terpan, 2015).

In contrast, quantitative studies assess the degree of legalization in international instruments through standardized coding frameworks, seeking to uncover variations in institutionalization, underlying determinants, and potential causal mechanisms. Prevailing quantitative approaches typically measure legalization by detecting the presence or absence of specific clauses within an international instrument. Lechner devised a comprehensive coding framework comprising 262 clause features and 126 observable indicators to identify elements indicative of legalization within preferential trade instruments. The study employed manual binary coding of treaty provisions (1 = present, 0 = absent) and utilized latent variable modeling to combine multiple observed indicators into a single latent factor representing the degree of legalization (Lechner, 2016). Similarly, Böhmelt and Butkute operationalized the concept of “legalization” as a set of observable quantitative indicators by identifying the presence or absence of five elements: legal bindingness, enforcement mechanisms, monitoring mechanisms, quantitative targets, and dispute settlement procedures, and used them to analyze states' self-selection effects (Böhmelt and Butkute, 2018). Furthermore, similar quantitative coding approaches have also been extensively employed across other domains, including international human rights law (Kim, 2019), international security law (Carpenter and Montgomery, 2020), and international dispute settlement (Weeramantry et al., 2023), demonstrating the methodological versatility of legalization measurement frameworks.

Focusing on the specific characteristics of each dimension of legalization, scholars have gradually developed a variety of targeted coding methods. The delegation dimension primarily assesses the existence and strength of vertical power distribution structures within international institutions and is therefore often operationalized as an ordinal coding scheme. Allee and Peinhardt classified the level of delegation into three categories (0 = no delegation, 1 = partial delegation, 2 = whole delegation). They manually coded each clause of the document to determine its specific delegation arrangements within dispute settlement mechanisms (Allee and Peinhardt, 2010). Pham and Thomson adopted a similar approach, using a binary classification (presence/absence of delegation) to manually code clauses, thereby measuring the degree of delegation to member states in the ASEAN instrument (Pham and Thomson, 2025). The precision dimension mainly evaluates the clarity of content expression by examining the wording structure and semantic specificity of the international instrument. Manger and Peinhardt constructed a legal wording dictionary to measure the precision of international instruments by calculating the frequency and proportion of linguistic features reflecting constraint, ambiguity, and flexibility in treaty clauses (Manger and Peinhardt, 2013). Similar to the precision dimension, the obligation dimension assesses the strength of rule bindingness and normative effectiveness by analyzing the use of modal structures in international instruments. For example, from a semantic perspective, Boginskaya measured the overall obligation strength of an international instrument by identifying the frequency, distribution, and semantic content of modal verbs expressing permission, obligation, and prohibition in legal discourse (Boginskaya, 2022).

### 2.2 Legal text analysis methods based on NLP techniques

Existing studies on international instruments generally employ cosine similarity–based text analysis methods to measure semantic similarity between clauses or documents. Lie proposed a computational framework called PITAD-Plus, which combines Jaccard distance, Levenshtein distance, and cosine similarity to examine textual similarity between clauses, identifying patterns of clause inheritance and reuse across treaties, and assessing states' influence in the processes of treaty innovation and linguistic diffusion (Lie, 2023). Uddin also adopted a similar approach, using TF-IDF vectorization combined with cosine similarity to evaluate the textual similarity of clauses among bilateral investment treaties, thereby assessing the relative dominance of states in shaping the dissemination and diffusion of treaty language (Uddin et al., 2024). Beyond similarity-based approaches, other studies have employed manually annotated legal corpora to train traditional supervised learning models for the automatic identification of key legal elements, such as definitions, obligations, and prohibitions, as well as for multi-label classification and topic mapping of legislative provisions (de Maat et al., 2010; Boella et al., 2012).

In recent years, as interdisciplinary research between NLP and law has advanced, LLMs have been increasingly applied to legal tasks. Multiple studies have demonstrated that, even under zero-shot conditions, LLMs exhibit strong capabilities for understanding and discriminating among legal texts, enabling them to accurately identify and classify normative linguistic expressions. In an extensive corpus study involving 18,614 contractual clauses, Sharma et al. demonstrated that several mainstream LLMs, including GPT-4, could accurately identify obligation-related clauses in contract texts under zero-shot conditions and achieved high accuracy across various classification categories (Singh et al., 2025). Similarly, in a study based on the CUAD dataset, Savelka and Ashley demonstrated that GPT-4 can robustly identify multiple types of contractual clauses in zero-shot classification tasks, with its performance in specific categories approaching that of traditional supervised models trained on large-scale, manually annotated corpora (Savelka and Ashley, 2023). Moreover, in research on automatic summarization of legal texts, Deroy et al. conducted comparative experiments and found that LLMs, even in weakly supervised or zero-shot settings, significantly outperformed traditional extractive summarization methods across multiple quality metrics (Deroy et al., 2025).

However, LLMs display pronounced limitations when confronted with more complex legal reasoning tasks. Blair-Stanek et al. found that although mainstream models such as GPT-4 can accurately identify and apply relevant statutes in statutory reasoning problems, they often produce inconsistent or even contradictory conclusions when case contexts change slightly (Blair-Stanek et al., 2023). Similarly, Guha found in studies on legal writing tasks that although LLMs can generate structurally complete texts under the IRAC framework (Issue–Rule–Analysis–Conclusion), they often exhibit problems such as logical leaps, factual confusion, or improper application of legal norms in the crucial “Analysis” section (Guha et al., 2023). These studies suggest that current LLMs have not yet developed stable and reliable jurisprudential reasoning capabilities, particularly when confronted with complex logical structures, overlapping rules, or conflicting norms; their reasoning accuracy and consistency remain insufficient to meet the professional standards of legal practice. This limitation is one of the primary reasons LLMs have yet to gain widespread adoption in empirical analysis of international legal instruments.

Moreover, the phenomenon of “hallucination” in LLMs presents a significant obstacle to their reliable application in the legal domain. Such issues are particularly prominent in legal text analysis tasks, as models often produce seemingly plausible yet unfounded interpretations of clauses, case matches, or legal conclusions (Ji et al., 2023). For instance, Dahl et al. found that mainstream LLMs exhibit error rates ranging from 58% to 82% in legal question-answering tasks, with specific models (such as Llama-2) reaching 88%. Even when guided by prompts for self-verification, the models' ability to detect and correct their own errors remains limited, making it challenging to identify and rectify inaccuracies reliably (Shao et al., 2025). Consequently, mitigating hallucination frequency and enhancing factual reliability remain central challenges for the practical application of LLMs in the empirical study of international legal instruments.

### 2.3 Enhancement strategies for LLMs

To mitigate LLMs' limitations in reasoning and hallucination problems in legal text analysis, researchers have proposed various enhancement strategies. One of the most prevalent approaches is fine-tuning, whereby models are further trained on domain-specific legal corpora to enhance their adaptability and predictive accuracy in legal contexts. In the legal field, the most representative pretrained model is Legal-BERT. Built upon the original BERT architecture, Legal-BERT is further pretrained on European Union legislative and case law corpora and serves as the foundational model for most contemporary legal NLP fine-tuning studies (Chalkidis et al., 2020). Models such as Legal Pro-BERT (Tewari, 2024), Recht BERT (Looijenga, 2024), and Case Law-BERT (Zheng et al., 2021) are all built upon Legal-BERT and achieve performance improvements on specific downstream law tasks through fine-tuning. In addition, other studies have proposed Lawformer, a model based on the Longformer architecture, which is better suited to handling the lengthy nature of Chinese judicial judgments and to optimizing fine-tuned model performance for long-document processing (Xiao et al., 2021). Multiple studies confirm that fine-tuning on specialized legal corpora markedly enhances LLM performance in legal text analysis and concurrently reduces hallucination frequency. However, due to the rapid evolution of legal norms, models must be frequently retrained to maintain semantic and knowledge currency. The high computational and labor costs significantly constrain the long-term sustainability of this approach (Shao et al., 2025).

The emergence of RAG technology has effectively alleviated the problem of models requiring frequent retraining to maintain the timeliness of knowledge. This approach introduces a dynamic semantic retrieval mechanism during the generation stage, enabling the model to access highly relevant external expertise to the input, thereby enhancing the interpretability of model outputs and effectively reducing hallucination phenomena (Lewis et al., 2020). Compared to fine-tuning methods, RAG can maintain model timeliness and adaptability by expanding and updating external knowledge bases, thereby offering advantages in resource efficiency and maintainability. In recent years, a growing body of research has employed RAG techniques to enhance the complex text-processing and reasoning capabilities of LLMs. For example, Siddharth and Luo proposed an explicit relation extraction method based on patent texts, which automatically identifies “entity–relation–entity” triples in engineering literature to construct a structured knowledge base for retrieval and reasoning, thereby providing factual constraints and content integration support for the LLM generation process (Siddharth and Luo, 2024). In the legal domain, Wiratunga et al. proposed the CBR-RAG framework, which introduces indexed vocabulary and similarity-based knowledge containers during the initial retrieval phase of the case-based reasoning loop to enhance LLM queries using contextually relevant legal cases (Wiratunga et al., 2024). Similarly, Matak and Chudziak introduced gAIus, a cognitive LLM-based agent architecture that retrieves task-specific legal knowledge to enhance model performance across legal reasoning and clause-retrieval tasks (Matak and Chudziak, 2025). Therefore, the RAG method provides a practical approach to addressing the reasoning deficiencies and hallucination issues of LLMs in complex legal reasoning tasks.

Moreover, prompt engineering constitutes a crucial technique for enhancing the reasoning and task-specific performance of LLMs. Existing studies have shown that even in zero-shot or few-shot scenarios, well-designed task prompts can significantly improve model performance on new tasks without requiring additional parameter training (Radford et al., 2019; Brown et al., 2020). Researchers have developed various advanced prompting strategies to enhance LLMs' logical reasoning capabilities in complex, multi-step problem-solving contexts. For example, Wei et al. proposed the Chain-of-Thought (CoT) prompting method, which explicitly guides models to generate step-by-step reasoning processes, improving accuracy on mathematical and commonsense reasoning tasks to 90.2% (Wei et al., 2022). Building upon this, Zhao et al. developed the “Logic-of-Thought” prompting strategy, incorporating an iterative “think–verify–revise” cycle to minimize reasoning errors. Experimental results show that this method outperforms CoT on both the GSM8K and AQuA datasets (Zhao et al., 2024). Moreover, derivative prompting frameworks, including Tree of Thoughts (Yao et al., 2023), Thread of Thought (Zhou et al., 2023), and Chain-of-Table (Wang et al., 2024), have likewise demonstrated efficacy in enhancing LLM performance on complex legal reasoning tasks. Therefore, it is evident that well-designed prompts play a crucial role in improving LLMs‘ legal text analysis capabilities.

### 2.4 Limitations of the current research

Although coding and analytical approaches to international instruments grounded in the legalization conceptual framework have grown increasingly sophisticated, and notwithstanding the considerable advances of NLP technologies within the legal domain, existing research continues to exhibit notable limitations across several critical dimensions.

First, existing legalization research still mainly relies on manual judgment; such methods not only entail high labor costs in implementation but also make it difficult to avoid consistency risks arising from coders' subjective judgments. Although certain studies have sought to address these shortcomings by introducing quantitative coding criteria, the resulting schemes have largely remained confined to binary classifications or coarse ordinal scales, and no universally applicable operational standard has thus far been established across the literature. These limitations have collectively undermined the capacity of existing research to conduct granular and continuous semantic measurement of international instrument content, while simultaneously imposing significant constraints on cross-issue comparative analyses of legalization. These limitations have, to a considerable extent, undermined the capacity of existing scholarship to perform continuous and structured semantic measurement of international instrument content, while also constraining the scope of comparative analysis of the degree of legalization across issue areas.

Second, although LLMs have made certain progress in legal text classification and reasoning tasks, their application in the specific task of scoring the degree of legalization of international documents remains relatively limited. In zero-shot or low-supervision settings, the inherent hallucinatory tendency of LLMs may pose risks to the stability of reasoning and the consistency of explanatory arguments in complex, multidimensional scoring tasks. However, the existing literature still pays insufficient attention to highly institutionalized text genres, such as international documents, and lacks a systematic discussion of mitigation mechanisms for the above risks in the context of international document analysis.

Third, existing studies have provided initial empirical evidence that enhancement strategies—such as fine-tuning, RAG, and prompt engineering—can improve the performance of large language models in legal text processing tasks. However, the application of these strategies to the specific task of assessing the degree of legalization in international documents remains underexplored. From a theoretical integration perspective, no prior study has systematically embedded the three-dimensional legalization framework proposed by Abbott et al. into prompt design, leading to a lack of scoring approaches that are both theoretically grounded and supported by traceable reasoning processes. In terms of data infrastructure, the field currently lacks publicly available, standardized benchmark datasets. This fundamentally constrains the effective application of LLM enhancement strategies, such as RAG and fine-tuning, in this task context and impedes systematic performance benchmarking.

In summary, existing research still has limitations in terms of the uniformity of coding standards, the degree of integration between theoretical frameworks and model reasoning mechanisms, and the application pathways of model enhancement strategies in specific task contexts. These limitations have collectively resulted in the fields of international law and international relations not yet having developed mature scoring tools that combine automation, scalability, and interpretive transparency to support the assessment of the degree of legalization of large-scale international instruments. To address the above research gaps, this paper proposes a hybrid scoring architecture that integrates CoT prompting and an RAG pipeline. This architecture uses the “five-level coding standard” and “step-by-step binary judgment logic” as the design basis for the prompt template, supplemented by a semantic vector database constructed through expert annotation as retrieval support, thereby effectively improving the reasoning stability and scoring consistency of LLMs in international instrument scoring tasks. Subsequent sections will systematically elaborate on the methodological design of this scoring framework.

## 3 Research methods

### 3.1 Overview of scoring framework

The objective of this study is to construct a hybrid scoring framework integrating RAG and CoT prompting mechanisms to assess the degree of legalization of international instruments. Compared with traditional RAG processes that rely on single-round semantic retrieval, this study introduces adaptive quality-weighted ranking and domain-specific secondary filtering mechanisms on the retrieval side to optimize the relevance and semantic matching accuracy of candidate clauses; On the generation side, this study embeds a CoT prompt template based on the legalization conceptual framework proposed by Abbott et al. into the prompt construction process, and enforces a stepwise decision procedure across three dimensions to strictly constrain the reasoning path of LLMs. The framework mainly consists of three stages: (1) construction of a vector-indexed clause database; (2) retrieval of similar exemplars; (3) automated reasoning and scoring.

#### 3.1.1 Stage one: construction of the vector-indexed clause database

The technical implementation of this scoring framework is predicated upon the construction of a vector-indexed clause database. First, clause texts containing substantive normative content are systematically extracted from the target regional international instruments and standardized into structured JSON text units, thereby ensuring consistency across subsequent coding and retrieval operations. Subsequently, legal experts systematically annotate each clause along the three dimensions of obligation, precision, and delegation, in accordance with the five-level coding standard and stepwise binary decision process proposed in this study. All annotated clauses are subsequently encoded into semantic vector representations via the text embedding model and ingested into a semantic retrieval-enabled vector database, where they are made available for invocation by the scoring pipeline. Based on the foregoing procedure, this study constructs a vector-indexed database comprising 2,611 clause units, which serves as the knowledge foundation for the operational deployment of the framework. It should be noted that the clause sources of this database are currently confined to ASEAN instruments; accordingly, its external validity and cross-regional generalizability with respect to instruments of other regional international organizations warrant further empirical investigation.

#### 3.1.2 Stage two: retrieval of similar exemplars

After completing the construction of the vector-indexed clause database, the scoring process formally begins. After the system receives the query clause, it first encodes it into a semantic vector representation using the text embedding model, then retrieves the top-20 semantically nearest clauses from the vector-indexed database using a dense vector similarity retrieval mechanism, constructing the initial candidate set. Subsequently, the system introduces a confidence-weighted re-ranking mechanism that re-ranks candidate clauses by integrating retrieval similarity scores and preset quality evaluation metrics, and selects the top-5 clauses with the highest comprehensive score as the refined candidate set. To ensure semantic matching accuracy, the system further employs a pre-trained cross-encoder to conduct a secondary semantic matching evaluation of the refined candidate set, and thereby determines the final set of exemplar clauses to be input into the prompt.

#### 3.1.3 Stage three: automated reasoning and scoring

The selected exemplar clauses are integrated into a CoT prompt template designed based on the legalization conceptual framework. This prompt template guides the model's reasoning through a programmatic stepwise decision process and sequentially evaluates the three dimensions of obligation, precision, and delegation. At the same time, the embedded base prompt imposes normative constraints on the model generation process by explicitly specifying reasoning steps and output format, thereby improving transparency and reproducibility of the results. Under the dual guidance of task instructions and reasoning prompts, the model generates scores for each query clause across the three dimensions of obligation, precision, and delegation (ranging from 0 to 1) and outputs the results in JSON format, with accompanying reasoning explanations.

To evaluate the performance of the proposed scoring framework, this study constructs two mutually independent test sets. The first test set comprises 254 expert-annotated clauses drawn from ASEAN documents and is used for in-domain evaluation; the second consists of 255 clauses from documents of other major regional organizations and is used to assess cross-domain generalization. In addition, four complementary evaluation metrics are employed to provide a comprehensive assessment of model performance. These include Quadratic Weighted Kappa (QWK), Mean Absolute Error (MAE), Spearman's rank correlation coefficient (ρ), and Exact Agreement Rate (EAR). Together, these four metrics capture scoring quality from multiple perspectives, including ordinal agreement, magnitude of deviation, rank-order consistency, and exact-match accuracy.

**Figure 1:**
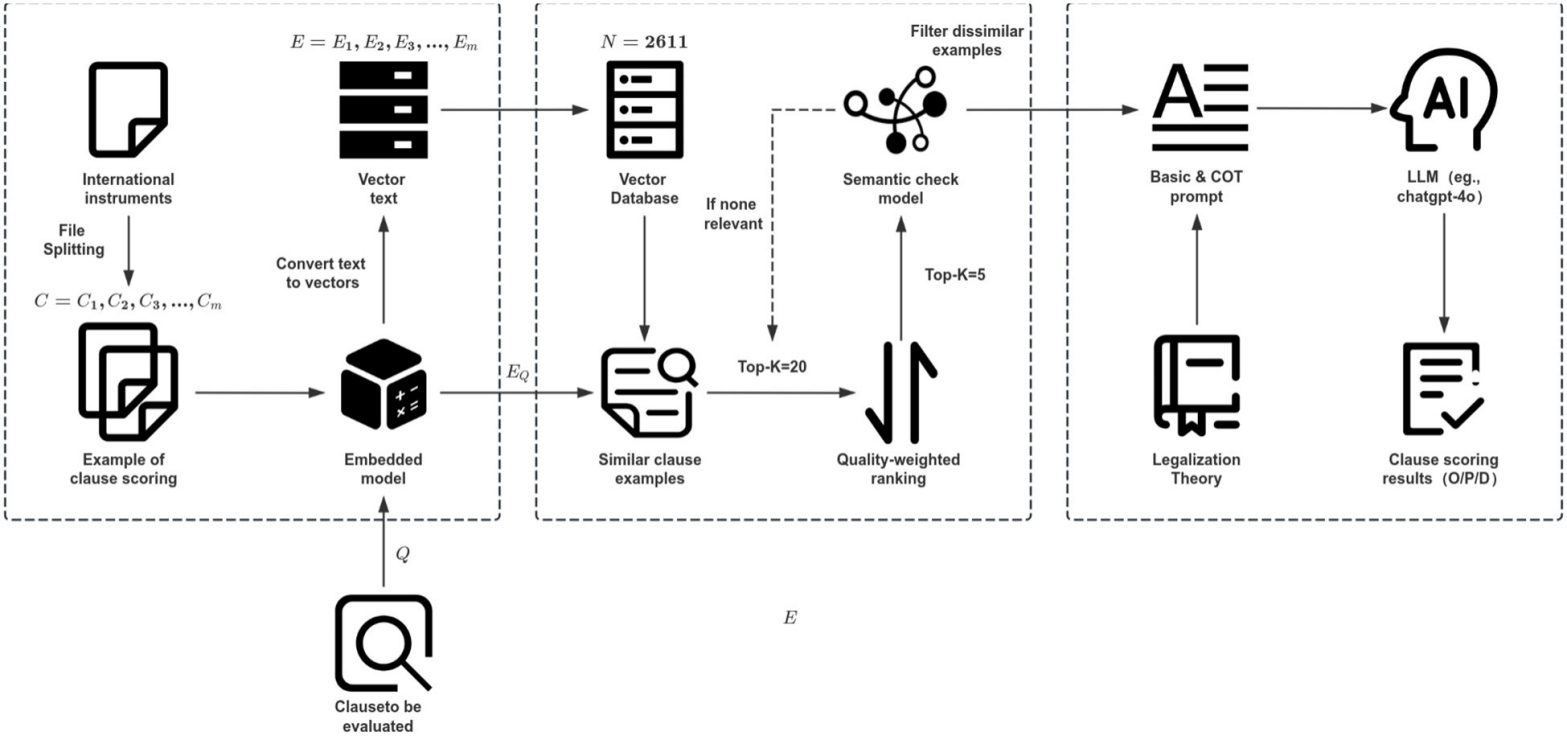
Schematic diagram of the hybrid RAG scoring framework based on CoT prompting.

Figure 1 presents the overall workflow architecture and core modular composition of the scoring framework proposed in this study. The framework constitutes a hybrid system that integrates expert rule systems, annotated data samples, and the execution capabilities of large language models, representing a concrete implementation of the “theory-driven + AI-execution” paradigm in legal text analysis. The framework follows the reasoning logic of human experts and, by incorporating stepwise reasoning mechanisms and semantically similar case-based retrieval, provides structured support and traceable explanatory pathways for model scoring decisions. Compared with purely data-driven models (such as Legal-BERT), which rely on statistical pattern learning and whose internal decision processes are difficult to audit directly, this framework explicitly externalizes the reasoning process and embeds domain knowledge in a structured form, thereby offering significant advantages in transparency and interpretability. This design orientation is particularly valuable in legal research and practice contexts that demand high levels of auditability and trustworthiness. The subsequent subsections provide detailed descriptions of the implementation and technical details of each functional module.

### 3.2 Definition and structure of the international instrument

International instruments refer to formal legal documents concluded by actors in the international community to establish, adjust, or affirm their mutual rights and obligations, institutional arrangements, and legal norms (Johnston, 2023). According to their legal binding force, international instruments are generally classified into legal and political instruments. The former possess formal legal effect, violations of which incur corresponding international responsibility and typically take the form of treaties, conventions, or protocols; the latter reflect political intent or policy commitment and, although lacking formal legal enforceability, often exert substantial normative and coordinative influence in international relations, such as declarations, memoranda of understanding, and action plans. A central objective of assessing the degree of legalization in international instruments is to determine whether their institutional nature aligns more closely with legal or political instruments.

**Figure 2:**
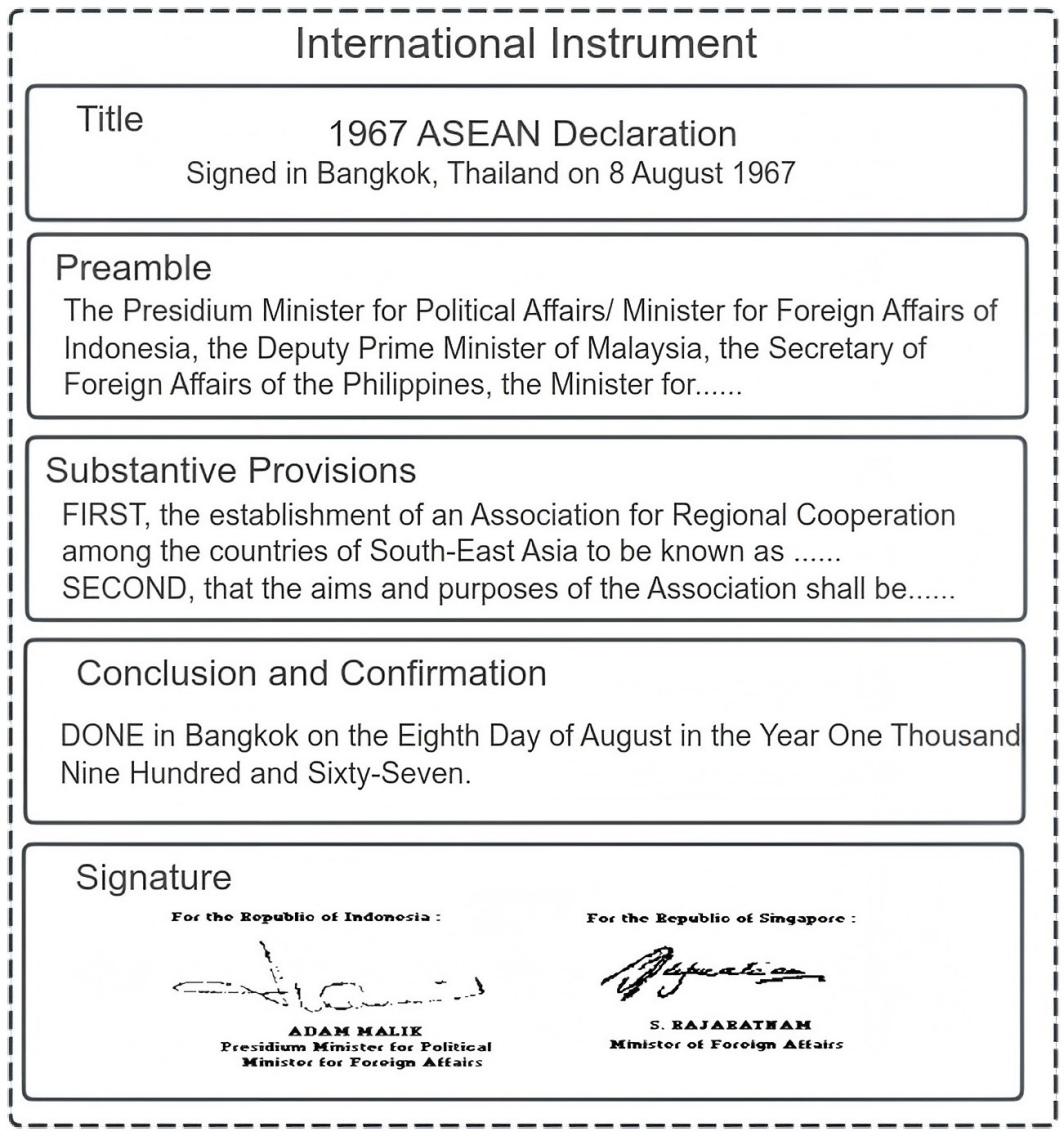
Schematic diagram of the structure of an international instrument.

Generally speaking, a complete international instrument comprises several basic components: title, preamble, substantive provisions, conclusion, confirmation, and signature sections. The specific structure is shown in Figure 2.

Notably, the different components of an international instrument contribute unevenly to its overall degree of legalization. The preamble, conclusion, confirmation, and signature sections primarily articulate political intent and normative context, fulfilling procedural and confirmatory functions that contribute mainly to the instrument's formal legitimacy and procedural validity. By contrast, the substantive clause section directly regulates the parties' rights and obligations, the design and implementation of oversight mechanisms, and the degree of specificity and clarity of legal provisions, thereby serving as the principal substantive basis for assessing the legalization of an international instrument. From the perspective of the functional logic of legal text structure, its status is, to some extent, similar to that of patent claims in patent documents—the latter define the scope of patent protection and constitute the main basis for determining legal validity and infringement (Siddharth et al., 2022). Accordingly, this study designates the substantive clauses as the primary analytical unit, ensuring that the legalization assessment focuses on the textual components most indicative of the three core dimensions of obligation, precision, and delegation.

### 3.3 Coding methods of legalization and CoT prompt templates

The core of understanding legalization lies in the dynamic variability of its three fundamental dimensions: obligation, precision, and delegation. This variability implies that international instruments do not conform to a simple binary of “legal” vs. “non-legal,” but instead exist along a continuum, exhibiting diverse characteristics in terms of institutional constraint, rule clarity, and modes of authority allocation (Abbott et al., 2000). When an international instrument is weakened in any of these dimensions, its overall degree of legalization tends to “soften.” Abbott and Snidal used the term “soft law” to describe such deviations from “hard law.” In extreme cases, when an international instrument entirely departs from formal legal constraint mechanisms and approaches purely political arrangements, it no longer possesses substantive characteristics of legalization (Abbott and Snidal, 2000). It is precisely this intrinsic continuity and diversity that make legalization a key analytical indicator for understanding and analyzing the institutional factors and evolutionary logic of international cooperation mechanisms.

To enable a more precise assessment of legalization across the dimensions of obligation, precision, and delegation, this study develops a five-point coding scheme based on the binary coding method proposed by Abbott et al., thereby enabling a finer-grained evaluation of clause-level legalization. In practice, this paper uses the binary judgment criteria commonly used in mainstream studies of international instruments as the basis for clause scoring, while designing a stepwise decision-making process to ensure the consistency and operability of results across dimensions. This coding scheme and decision process are further embedded in the CoT prompt template to guide LLMs in performing step-by-step reasoning during clause-scoring tasks, thereby enhancing the logical consistency and interpretability of model judgments. The subsequent section elaborates on the conceptual design and operational logic of the coding scheme and decision process for each of the legalization dimensions.

#### 3.3.1 Obligation dimensions

Obligation refers to the binding behavioral requirements that states or other international actors are expected to fulfill under specific rule systems or political commitments. The degree of obligation in an international instrument primarily depends on the normative nature expressed in its clauses, whether the rule is mandatory, merely recommendatory, or permissive with a degree of discretion. With respect to specific linguistic instantiation, formulations such as “Member States shall take necessary legislative measures…” indicate mandatory obligations. By contrast, “Member States should cooperate to promote…” typically carries a recommendatory character. Whereas, “Member States may take appropriate measures…” signals the permissive normative character of the provision, conferring upon relevant actors a defined degree of discretionary latitude. It can thus be seen that the linguistic formulation of clauses, and in particular the selection and use of modal verbs, constitutes the core linguistic indicator for determining the nature and intensity of legal obligations (Rajamani, 2016). Building on this reasoning, Abbott et al. (2000) further divided the obligation dimension into a six-level conceptual scale, ranging from unconditional mandatory obligations to non-legal norms that explicitly deny legal intent. This classification intuitively reveals the continuum of the obligation dimension from highly binding legal commitments to weakly binding political undertakings. It has been widely applied in subsequent empirical research on the measurement and coding of legalization in international instruments (Böhmelt and Butkute, 2018; Schulte and Carolan, 2024).

However, the hierarchical classification of the obligation dimension advanced by Abbott et al. focuses primarily on the existence and binding intensity of obligations, without considering the attribution of legal responsibility or the institutional consequences that may result from violations of mandatory provisions. In general, for states or other international actors to assume international obligations is to subject their conduct to constraint and oversight within the institutional framework constituted by the normative principles, procedural mechanisms, and regulatory discourse of international law. If such defined obligations are violated, the consequences extend beyond reputational loss at the political level and may trigger formal international responsibility mechanisms, including sanctions, reparations, and various forms of legal remedies (Köchler, 2019). In designing the obligation dimension coding for trade instruments, Lechner specifically incorporated the “enforceability of obligation violations” into consideration. Her coding scheme uses “whether a member's violation of non-trade obligations triggers a trade sanctions mechanism” as a key criterion for assessing post-obligation conditions, thereby introducing an institutional enforcement and compliance perspective into the measurement of the obligation dimension (Muhammad, 2022). In view of this, the obligation dimension scoring criteria constructed in this study take into account two levels of consideration in their operational design: First, whether the actors are bound by the normative requirements established by the provision; Second, the legal responsibility or institutional consequences that may arise from violations of the relevant provisions. The five-level coding standard for the obligation dimension is detailed in Table 1.

**Table 1:** Coding scheme for each level of the obligation dimension.

Score	Description
1.0	The clause clearly stipulates the legal obligations that contracting parties shall undertake, and includes specific consequences for breach, such as legal responsibility, sanctions, penalties, disqualification, or loss of rights.
0.75	The clause clearly stipulates the legal obligations that contracting parties shall undertake, but does not include any consequences for breach.
0.5	The clause clearly stipulates legal obligations to be undertaken, but exceptions exist; or the subject of the obligation is not the contracting parties (for example, the Secretariat or a working group).
0.25	The clause only expresses political commitments or intentions to cooperate (such as “shall endeavor,” “encourage,” or “promote”), and does not constitute a legal obligation.
0.0	The clause contains neither obligations nor political commitments, and is limited to vision statements, background, definitions, or procedural descriptions.

Based on the above coding standards for each level, this study designs a stepwise decision-making process grounded in binary logic to ensure accurate and consistent scoring within the obligation dimension. The detailed procedural framework is presented in Table 2.

**Table 2:** Stepwise binary decision process of the obligation dimension.

Step	Question	Scoring rule
1	Does the clause contain any normative statements (legal obligations or political commitments)?	No → 0.0 Yes → step 2
2	Does the clause contain only political commitment statements?	No → step 3 Yes → 0.25
3	Are the legal obligations stipulated in the clause subject to exceptions, or is the obligation-bearing entity not the contracting parties?	No → step 4 Yes → 0.5
4	Do the legal obligations stipulated in the clause include specific consequences for breach?	No → 0.75 Yes → 1.0

This process examines the binding force of clauses on actors' conduct and the potential legal and institutional consequences of their violation, thereby categorizing and mapping the obligation dimension into five observable levels.

#### 3.3.2 Precision dimensions

Precision refers to whether the rules governing the behavioral requirements, scope of application, and conditional arrangements for states or other international actors are clear, specific, and unambiguous. For example, if a clause provides that “Contracting Parties shall complete a 20% reduction in greenhouse gas emissions by 31 December 2025,” clearly specifying the subject, action, time, and corresponding implementation details, it constitutes a highly precise normative formulation; conversely, if it merely states that “Parties shall appropriately take measures to promote environmental protection,” without specifying any implementation details, it reflects a lower level of normative precision. However, some scholars have noted that excessive precision in legal norms may create the risk of expressio unius est exclusio alterius, meaning that the expression of one thing implies the exclusion of others. While precise formulations improve interpretability and legal clarity, they may inadvertently constrain the scope of applicability, creating loopholes for obligation avoidance (Percy, 2007). For this reason, precision is often regarded as the dimension exerting relatively less influence on legalization. Based on the clarity of normative content, (Abbott et al., 2000) divided the precision dimension into a five-level conceptual scale, ranging from highly determinate rules to those in which it is impossible to assess whether behavior conforms to requirements.

However, Abbott et al.'s definition of the clarity of normative content is relatively broad and lacks specific, empirically operationalizable assessment criteria. To address this deficiency, this study draws on a commonly used precision measurement method in quantitative international law research, namely word frequency analysis. This method relies on dictionaries of vague words and qualifiers, and evaluates the overall clarity of the text by calculating the frequency and relative proportion of vague words, modal verbs, and conditional clauses in the instrument (Grimmer and Stewart, 2013). However, this study does not directly adopt the above word frequency analysis method; rather, it distills its analytical approach, focusing on textual linguistic features, and extends it to semantic and structural analysis at the clause level to capture more fine-grained precision differences in the text. Specifically, this study evaluates whether clauses employ clear, operationalizable language to define actors, temporal conditions, behavioral patterns, and substantive content, serving as the core basis for determining textual clarity. The more specific and explicit a clause's provisions regarding the above implementation details, the less discretionary space is available to actors in the implementation and interpretation process, and the higher the corresponding precision. Conversely, clauses with vague or non-operationalizable formulations will expand the interpretive space, resulting in a corresponding decrease in precision. The five-level coding standard for the precision dimension is detailed in Table 3.

**Table 3:** Coding scheme for each level of the precision dimension.

Score	Description
1.0	The clause clearly specifies the subject, specific action, implementation method, timeline or frequency, and target object. The wording is clear, unambiguous, and directly operational.
0.75	The clause clearly specifies the subject and specific action but lacks one or more key implementation details (such as method, time, or object). The wording is relatively vague and less operational.
0.5	The clause only states the subject and specific action, lacking key implementation details (such as method, time, and object). It merely expresses intent and lacks clear operability.
0.25	The clause does not clearly specify the subject or action and adopts directional or open-ended language.
0.0	The clause does not contain any directive content and is limited to background, principles, or value declarations.

Based on the above coding standards for each level, this study designs a stepwise decision-making process grounded in binary logic to ensure accurate and consistent scoring within the precision dimension. The detailed procedural framework is presented in Table 4.

**Table 4:** Stepwise binary decision process of the precision dimension.

Step	Question	Scoring rule
1	Does the clause contain any directive content?	No → 0.0 Yes → step 2
2	Does the clause clearly specify the subject and specific action?	No → 0.25 Yes → step 3
3	Does the clause only state the subject and specific action while omitting all key implementation details?	No → step 4 Yes → 0.5
4	Does the clause clearly specify all key implementation details?	No → 0.75 Yes → 1.0

This process evaluates the textual clarity of each clause, categorizing and mapping the precision dimension into five empirically observable levels.

#### 3.3.3 Delegation dimension

Delegation refers to the extent to which states or other international actors transfer or entrust the authority to interpret, adjudicate, or implement instruments to third-party institutions or mechanisms. Abbott et al. (2000) distinguish delegation into two primary forms: delegation of dispute settlement authority and delegation of rule-making and implementation functions. The former entails the conferral of interpretative and adjudicatory authority upon third-party entities such as international courts or arbitral tribunals. In contrast, the latter concerns the delegation of rule-making, amendment, and implementation functions to specialized institutions. Only when a clause explicitly grants the authority of dispute interpretation, adjudication, or enforcement to an independent third-party institution or mechanism, and recognizes that its decisions have final and binding effect, can it be regarded as an institutional arrangement with the highest degree of delegation. For example, “Any dispute arising from the interpretation or application of this Agreement shall be submitted to an independent arbitral tribunal for adjudication, whose award shall be final and binding upon the contracting parties.” Conversely, if the relevant authority is entirely retained within the contracting parties and is not transferred or delegated to any third-party institution, the degree of delegation of the institutional arrangement is at the lowest level. For example, “The contracting parties shall resolve disputes through consultation.” Based on this, Abbott et al. (2000) operationalized dispute-settlement delegation and rule-implementation delegation as four-level and five-level conceptual scales, respectively, to characterize hierarchical differences in authority allocation and enforcement mechanisms within international arrangements.

Although the categorization of the delegation dimension proposed by Abbott et al. provides essential insights for the quantitative coding of international instrument legalization, its classification system suffers from an equivalence problem. In their indicator system for the delegation dimension, the authors treat dispute-settlement delegation and rule-making/implementation delegation as parallel elements, implicitly assuming that both contribute equally to the degree of legalization in international instruments. However, in practice, the binding force arising when states or other global actors delegate interpretive and adjudicative authority over established rules to independent third-party institutions is generally much more potent than that resulting from granting rule-making and implementation powers to bodies (Bélanger and Fontaine-Skronski, 2012). The root of this phenomenon lies in the fact that rule-making and implementation delegation in international instruments typically grant functions to collective decision-making bodies composed of representatives from contracting parties, rather than to third-party institutions independent of the parties, thereby limiting their binding effect. Koremenos introduced the concept of internal delegation to describe arrangements in which authority is retained within member states or within intergovernmental bodies composed of their representatives. Therefore, these two types of delegation mechanisms do not exert equivalent effects in enhancing the legalization of international instruments (Koremenos, 2008). Based on the above analysis, this study incorporates the conceptual distinction between internal and external delegation into the design of the delegation dimension scoring rubric. The degree of delegation embodied in each provision is assessed by examining, as the primary determinative criteria, whether authority is transferred to an independent third-party institution or mechanism, and whether the delegation arrangement possesses substantive enforceability. The five-level coding standard for the delegation dimension is detailed in Table 5.

**Table 5:** Coding scheme for each level of the delegation dimension.

Score	Description
1.0	The clause grants an institution adjudicative and enforcement functions over state behavior with direct legal effect. These powers take effect automatically.
0.75	The clause grants an institution adjudicative and enforcement functions over state behavior with direct legal effect, such as dispute settlement, obligation enforcement, sanctions, compulsory supervision, issuing binding interpretations, or making final non-appealable decisions, but their application is subject to specific conditions, such as requiring consent of contracting parties, procedural triggers, or requests; or the institution is granted administrative functions that may play a decisive role in instrument implementation, such as approval/veto power, rule-making authority, budget allocation authority, or certification authority.
0.5	The clause grants an institution administrative functions. Such functions do not play a decisive role in instrument implementation.
0.25	The clause does not mention a formal institution, but designates specific contracting parties to undertake administrative functions, including coordination, technical assistance, information collection, and advisory provision. Such functions do not play a decisive role in treaty implementation.
0.0	The clause does not mention any institution or contracting party; or although mentioned, no specific functional role or responsibility is assigned.

Based on the above coding standards for each level, this study designs a stepwise decision-making process grounded in binary logic to ensure accurate and consistent scoring within the delegation dimension. The detailed procedural framework is presented in Table 6.

**Table 6:** Stepwise binary decision process of the delegation dimension.

Step	Question	Scoring rule
1	Does the clause assign any function to an institution or contracting party?	No → 0.0 Yes → step 2
2	Is the clause limited to assigning specific functions to contracting parties (rather than a formal institution)?	No → step 3 Yes → 0.25
3	Do the functions assigned to the institution constitute non-decisive administrative functions?	No → step 4 Yes → 0.5
4	Do the functions assigned to the institution constitute decisive administrative functions or conditional adjudicative and enforcement authority?	No → 1.0 Yes → 0.75

This process evaluates both the form of authority delegation and its enforceability, categorizing and mapping the delegation dimension into five empirically observable levels.

### 3.4 Data selection and preparation

Regarding data selection and processing, this study uses ASEAN legal instruments as the primary corpus. Through a structured text preprocessing pipeline and domain-expert annotation, this study constructs a vector-indexed clause database to support automated scoring of the legalization level of international instruments. Serving as the foundational module of the overall system architecture, this database furnishes a reliable data infrastructure for the subsequent RAG pipeline and legalization-level analysis.

#### 3.4.1 Data collection

This study employs the ASEAN instrument as the foundational corpus for developing the proposed scoring framework. Compared with international organizations such as the European Union, which possesses tens of thousands of legal and political instruments, the number of ASEAN instruments is relatively moderate, making it a more suitable starting point for quantitative legalization measurement and methodological validation. The research team sourced publicly available ASEAN instruments from the center for International Law (CIL) Database at the National University of Singapore^1^ This database covers ASEAN documents from its founding to the present, containing approximately 1,772 international instruments signed by its member states. To ensure the representativeness and diversity of content in the corpus, this study adopted a combined strategy of random sampling and type coverage, selecting 73 ASEAN instruments of various types as the corpus foundation for building the vector-indexed clause database.

#### 3.4.2 Data preprocessing

During the data preprocessing stage, the research team established a standardized workflow to ensure the dataset's structural, formatting, and content consistency. The preprocessing procedure comprised the following steps:

1. Non-textual elements, including headers, footers, and page numbers, were removed, and redundant blank lines and spaces were normalized to single spaces to ensure clean and consistent formatting.
2. Based on the structural regularities of the ASEAN instrument, a series of regular-expression rules was developed and implemented to extract the substantive clause sections from each document automatically.
3. For structurally irregular or non-standard documents, manual segmentation and expert verification were conducted to ensure semantic integrity and accuracy of text unit division.
4. Four core data fields, id (unique identifier), document_title (title), year (publication year), and text (clause content) were established to guide metadata extraction and structured data mapping. All clause-level text units were standardized and stored in JSON format, with each clause represented as an independent data object, facilitating subsequent retrieval and model interaction.
5. Clause completeness, field consistency, and metadata accuracy were systematically verified to ensure overall data reliability and integrity.

Through the above standardized processing workflow, this study transformed the original ASEAN document texts into a complete, standardized, and traceable corpus resource, thereby providing a high-quality data foundation for subsequent vector database construction.

#### 3.4.3 Data annotation

Following the completion of semantic boundary detection and clause segmentation as preprocessing procedures, legal experts manually annotate the resulting clause-level textual units in accordance with the previously established five-level coding standard and sequential binary decision process. All legal experts participating in the annotation have a background in international law, have long been engaged in research on international institutions and treaty text analysis, and are familiar with the three-dimensional legalization framework proposed by Abbott et al. Before formal annotation, the research team conducted multiple rounds of discussion and small-scale pilot annotations, using a unified coding guideline to ensure a shared understanding of the scoring criteria among all experts. To ensure the objectivity and independence of the annotation process, this study assigned two legal experts to annotate the same dataset and used the quadratic weighted QWK metric to evaluate inter-annotator agreement. The analysis results show that the QWK value between the two annotators is 0.804, indicating a high degree of consistency in their annotations, according to prevailing academic standards. The above results provide empirical support for the five-level coding standard and the step-by-step decision process developed in this study, indicating that the scoring framework has high inter-rater reliability and operational reproducibility in the normative scoring of international legal instruments.

Upon completion of the scoring phase, a disagreement-resolution mechanism was implemented to reconcile annotation discrepancies between the two legal experts. Specifically, if the score difference for a given clause was less than 0.5, the two annotators jointly reviewed the case and reached a consensus through discussion to determine the final score. When the score difference was equal to or greater than 0.5, a third expert was introduced to conduct an arbitration review to determine the final score.

In addition, this study introduces a local confidence metric for each clause's single-dimension score to intuitively measure the degree of divergence between the two experts' evaluations. The calculation formula is as follows:


$$\begin{array}{l} \text{Conf}=1-|{\text{L}}_{1}-{\text{L}}_{2}| \end{array}$$

In this formula, **L**_1_ and **L**_2_ denote the scores assigned by the two legal experts to the same clause within a specific dimension. The smaller the numerical difference between the two values, the higher the corresponding local confidence, indicating stronger consistency and stability in the experts' judgments for that dimension. These confidence metrics **Conf** are incorporated as weighting factors within the subsequent quality-weighted adaptive retrieval module, enhancing both the precision and robustness of the retrieval outcomes. After completing the expert annotation and disagreement reconciliation process, this study ultimately constructed a high-quality annotated dataset containing 2,611 clause-level text units. All the datasets and annotation guidelines are available at GitHub^2^.

### 3.5 Retrieval-augmented generation

In terms of retrieval mechanism design, this study constructed a vector database based on 2,611 expert-annotated clause units to support the semantic retrieval and contextual matching modules in the RAG process. Furthermore, the retrieval framework integrates dense vector search with a locally confidence-weighted re-ranking strategy to enhance semantic matching precision. It employs a Legal-BERT cross-encoder validation model for relevance classification and filtering, ensuring rigorous quality control throughout the retrieval-augmentation process.

#### 3.5.1 Vector database construction

This study utilizes an annotated dataset containing 2,611 clause units in the semantic vectorization process to generate vector embeddings for subsequent semantic retrieval and matching. For efficient text semantic embedding, this study employs the e5-large-v2 model (Wang et al., 2022). The model is built on the e5 framework and can generate high-quality 1024-dimensional dense semantic vectors that balance semantic similarity and vector representation accuracy. It exhibits excellent performance and precision in various semantic similarity tasks while maintaining high computational efficiency and scalability. The generated vectors are L2-normalized and stored in the Chroma DB vector database, facilitating efficient retrieval and management. This database enables efficient retrieval via a high-dimensional approximate nearest-neighbor structure, using cosine similarity as the primary metric for computing semantic similarity between vectors. Serving as the foundational layer of the RAG pipeline, this vector database enables efficient knowledge retrieval and access, thereby supporting the automated clause scoring and model evaluation stages of the framework.

#### 3.5.2 Retrieval and ranking strategy

To ensure the efficiency and stability of the RAG process in the automatic clause-scoring task, this study proposes and implements a multi-stage composite retrieval strategy. The strategy integrates dense vector retrieval with a local-confidence-weighted re-ranking mechanism, allowing the model to capture deep semantic correlations among clauses with high precision. By balancing matching accuracy and semantic relevance, this method significantly improves the overall quality of retrieval results and enhances the system's robustness and generalization in complex legal text contexts. The specific implementation process is as follows:

First, the system takes the text to be scored as query input, with each input unit corresponding to an independent clause in the international instrument. For example, the clause “The Contracting Parties shall, in accordance with their respective national laws, cooperate in preventing transnational crimes” is uniformly prefixed with “query:” per the e5-large-v2 model's technical specifications for asymmetric retrieval tasks. Subsequently, the system encodes the processed text into high-dimensional dense semantic vectors and performs similarity retrieval in the ChromaDB vector database. Each query returns the top 20 candidate clauses ranked by semantic relevance, along with semantic distance, text content, and associated metadata. The formula for calculating semantic similarity between vectors is as follows:


$$\begin{array}{l} {\text{sim}}_{\left(\text{p},\;\text{q}\right)}=\frac{{\text{V}}_{\text{p}}{•\text{V}}_{\text{q}}}{\left\|{\text{V}}_{\text{p}}\|\|{\text{V}}_{\text{q}}\right\|} \end{array}$$

Among these, **sim**_(**p, q**)_ represents the semantic similarity between the query clause and the candidate clause, calculated by the cosine similarity function from the dense semantic vector **V**_**q**_ of the query clause and the candidate clause vector **V**_**p**_. After completing the preliminary semantic retrieval, the system performs classification confidence evaluation on each candidate clause across three legalization dimensions and calculates the average confidence **Conf**_**avg**_ of the three. This metric, together with the vector semantic similarity, jointly constitutes the composite weighting factor, used to generate the composite quality score **S** of the candidate clauses, achieving re-ranking of the initial retrieval results:


$$\begin{array}{l} \text{S}={\text{sim}}_{\left(\text{p},\;\text{q}\right)}+{\text{Conf}}_{\text{a}v\text{g}} \end{array}$$

Finally, the system sorts all candidate clause units in descending order according to the composite quality score **S**, and extracts the top five clauses (Top-K = 5) as in-context learning examples, which are input into the large language model to guide score generation. This filtering mechanism, by applying dual-quality filtering to candidate samples, ensures the semantic relevance of the exemplars while accounting for their legal normativity, thereby improving the reliability and interpretability of the scoring results.

#### 3.5.3 Filtering and quality control of retrieval results

Although the vector database constructed in this study contains 2,611 annotated examples, its overall scale remains relatively limited, making it difficult to provide sufficiently comprehensive and semantically matched reference samples for all clause types. To reduce the risk of irrelevant examples being incorporated into the prompt template, this study introduces a Legal-BERT cross-encoder validation mechanism during the retrieval stage to classify and filter candidate clauses based on relevance (Ji et al., 2023). The model is built on the BERT architecture and has been domain-pretrained on a large-scale legal corpus. Owing to its deep adaptation to legal linguistic structures, the model effectively captures domain-specific terminology and logical reasoning patterns, exhibiting superior discriminative performance in clause-level semantic matching tasks. The detailed implementation procedure is outlined as follows:

The system initially retrieves the top 20 candidate clauses from the vector database, applies weighted re-ranking according to their composite quality scores, and selects the top five (Top-K = 5) as high-confidence candidate exemplars. The setting of the Top-k parameter is mainly based on common design principles of a two-stage retrieval architecture. In the initial dense retrieval stage, a relatively loose candidate size needs to be set to ensure a high recall rate (Karpukhin et al., 2020). Considering the size of the vector database in this study, the initial stage selects Top-20 clauses as the candidate pool to reduce the risk of missing potentially relevant clauses and control computational cost; In the final filtering stage, a balance needs to be achieved among semantic precision, contextual diversity, and prompt length constraints (Liu et al., 2024). Based on the distribution of clause lengths, the final selection chooses the top 5 clauses as examples to reduce information noise caused by irrelevant context.

Subsequently, the original query clause and each candidate clause are concatenated into a single input sequence and fed into the Legal-BERT model for semantic relevance classification, identifying the most relevant examples to the target clause. The cross-encoder performs interactive semantic modeling of the concatenated sequence using a self-attention mechanism, and attaches a linear classification layer to the (CLS) vector to output a relevance score s; Finally, the model maps the score through a Sigmoid activation function to a probability value within the (0,1) range and compares it with the threshold τ (τ = 0.5) to determine the binary semantic relevance between clause pairs. When all candidate clauses in a given batch are determined to be semantically irrelevant, the system automatically proceeds to the next round of screening until the entire candidate set has been fully traversed. If no retrieved example satisfies the predefined relevance threshold, the system omits sample injection into the CoT prompt template and instead directly leverages the LLM's zero-shot reasoning capability to execute the clause scoring process.

Furthermore, to effectively prevent the scoring framework from potential prompt injection and data poisoning risks during operation, this study designs a three-layer protection mechanism across the input, reasoning, and output stages:

1. Prompt-level safeguard: explicit behavioral constraints are incorporated into the prompt template, instructing the model to treat all retrieved passages strictly as legal text and to ignore any imperative or executable instructions, thus ensuring semantic integrity and alignment with the task objective.
2. Inference-level safeguard: during model execution, explicit safety constraints are passed via system-level messages, instructing the model to disregard any imperative or control-oriented language within clause texts, thereby preventing prompt injection from affecting the scoring process.
3. Output-level safeguard: during the output phase, all JSON-structured results and corresponding score sets returned by the model undergo rigorous format and numerical validation. When the system detects abnormal or non-compliant outputs, it automatically triggers a secondary scoring process to reduce potential interference from malicious generations or anomalous samples in subsequent statistical analyses.

## 4 Experimental setup and evaluation

### 4.1 Test dataset

To verify the reliability and external validity of the scoring framework, this study independently constructs two test sets of clause units, with no overlap with the vector database samples. The first test set is derived from the ASEAN instrument database, contains 254 clause units, and is used to evaluate the stability of the framework across the same-domain instruments. The second test set integrates the instrument corpora of five major regional international organizations—the European Union, the African Union, the Arab League, the Shanghai Cooperation Organization, and the Organization of American States—containing 255 clause units to examine the framework's cross-domain generalization capability. Both test sets adopt the same standardized preprocessing procedure described earlier and were independently annotated by two legal experts using the same five-level coding standard and stepwise binary judgment logic. Statistical analysis results show that the QWK values for the two datasets are 0.8235 (ASEAN test set) and 0.8092 (cross-domain test set), respectively, indicating high consistency between annotators' results. After the dispute resolution mechanism reconciled the divergent clauses, this study finally established two independent test sets.

### 4.2 Model selection and experimental configuration

This study selects four representative large language models released by OpenAI as experimental subjects, covering the complete capability gradient from lightweight to flagship: GPT-3.5-Turbo, GPT-4o-mini, GPT-4o, and GPT-5.2^3^. The above models exhibit progressive stratification in terms of parameter scale, architectural complexity, and logical reasoning capability, which facilitates a systematic examination of the association between model capability and clause-scoring accuracy. Regarding experimental access, all models are called via the official OpenAI API to ensure a consistent calling environment. For the experimental parameter configuration, the Temperature value for all models is set to 0 to eliminate uncertainty introduced by random sampling. Each experimental group also maintains strict consistency in the base prompt template design and output parsing logic to minimize the interference of confounding variables on experimental conclusions.

To examine the differential contributions of each core component in the scoring framework to overall performance, this study adopts a stepwise ablation analysis to construct the experimental scheme, designs four groups of controlled conditions, and quantitatively evaluates the marginal utility of each core module by sequentially removing them.

#### 4.2.1 Condition one: full framework (FULL)

Serving as the upper-bound reference for overall performance, this experimental group fully integrates the three core modules: base instructions, RAG retrieval, and CoT reasoning. Among these, the base instruction module defines the model identity, scoring dimensions, and output format. Building on this, the RAG component retrieves high-quality, semantically relevant annotated exemplars from the vector database for the clause to be scored, providing the model with contextual knowledge and a reference value. The CoT prompt template further guides the model to execute stepwise, structured scoring along the preset reasoning path, thereby enhancing the logical coherence and interpretability of the output results.

#### 4.2.2 Condition two: ablation of retrieval module (BASE + CoT)

This experimental group completely removes the RAG retrieval component; the model performs scoring in a zero-shot manner, relying solely on base instructions and CoT prompts, without external retrieved exemplars. The purpose of this experimental group is to independently evaluate the contribution of the external knowledge augmentation mechanism to the framework's overall performance.

#### 4.2.3 Condition three: ablation of reasoning module (BASE + RAG)

This experimental group completely removes the CoT prompt template; the model performs scoring solely on base instructions and clause-annotation exemplars retrieved by RAG, without structured reasoning guidance. The purpose of this experimental group is to independently evaluate the contribution of structured logical reasoning to the overall performance of the framework.

#### 4.2.4 Condition four: baseline condition (BASE)

As the global performance benchmark, this experimental group invokes the original language model using only base instructions, without applying any retrieval or reasoning enhancement measures. The purpose of this experimental group is to evaluate the model's intrinsic clause scoring capability under unaided conditions.

To ensure transparency in the experimental procedure, this study presents the prompt structure designs for the three modules—base instructions, CoT prompts, and RAG retrieval—in the Appendix for methodological verification and experimental replication. The complete prompt structures are shown in Appendix Figures A1–A3.

Furthermore, this study incorporates two categories of traditional supervised learning baselines to establish a comparative reference framework and comprehensively assess the applicability boundaries of the various methods examined on the international instrument clause-scoring task.

##### 4.2.4.1 Traditional machine learning baseline

This study selects TF-IDF vectorization combined with a logistic regression classifier as the statistical baseline model. This method is a classic text classification benchmark paradigm in the NLP field and is widely used to establish baselines for legal tasks due to its simple implementation, strong interpretability, and low computational resource requirements (Yuan et al., 2024). For feature engineering, this study uses a combined unigram and bigram strategy to extract text features, with a maximum feature dimensionality of 200,000. The classifier is trained using multinomial logistic regression, with class-balancing enabled (class_weight = balanced) to mitigate imbalance in the sample distribution across scoring tiers in the annotated data. Independent classifiers are trained for each of the three scoring dimensions (obligation, precision, and delegation) using the same training data as the 2,611-annotated clause dataset employed in the vector database construction phase described earlier. The purpose of introducing this baseline is to quantify, from a comparative perspective, the performance ceiling and inherent limitations of traditional frequency-based statistical methods in capturing the deep semantic structure of legal language.

##### 4.2.4.2 Fine-tuned pre-trained language model baseline

This study selects Legal-BERT, a pre-trained language model specialized for the legal domain, as the deep learning reference baseline. This model is a BERT variant that has been continually pre-trained on a large-scale legal corpus, with its vocabulary and language model specifically adapted to the specialized terminology and syntactic structures of legal texts, making it one of the representative supervised learning benchmarks for legal text classification tasks (Chalkidis et al., 2020). This study constructs a multi-task regression output architecture on top of the Legal-BERT encoder, with independent linear regression output layers for each of the three scoring dimensions (obligation, precision, and delegation), and employs the Smooth L1 loss function for end-to-end fine-tuning of all model parameters. The specific training configuration is as follows: learning rate of 2 × 10^−5^, per-device batch size of 4, gradient accumulation steps of 2 (equivalent global batch size of 8), training epochs of 6, and weight decay coefficient of 0.01; mixed precision training (fp16) is enabled in CUDA-supported hardware environments to improve training efficiency. The training data is consistent with the TF-IDF baseline, both using the dataset containing 2,611 annotated clauses. The purpose of introducing this baseline is to evaluate the upper bound of performance for domain-adapted supervised fine-tuning methods and to provide a methodological contrast with the zero-shot reasoning pathway of the present framework.

In summary, this study designs a total of six experimental conditions, with the overall experimental scheme structured around an evaluation strategy that integrates ablation analysis with supervised baseline comparison. Of these, four ablation experiments target the core functional modules of the proposed framework, quantifying the marginal contribution of each component to overall scoring performance by systematically removing the RAG retrieval component and the CoT reasoning module. The remaining two conditions constitute supervised learning reference systems, incorporating TF-IDF combined with logistic regression and Legal-BERT as the statistical and deep learning baselines, respectively, to evaluate the empirical performance gains of the proposed framework's zero-shot inference pathway relative to traditional supervised fine-tuning paradigms. The core configuration differences across the six experimental conditions are summarized in Table 7.

**Table 7:** Summary of experimental conditions and model configurations across six comparative settings.

Condition	Base Prompt	CoT	RAG	Backbone	Training
FULL	Yes	Yes	Yes	GPT series	Zero-shot
CoT	Yes	Yes	No	GPT series	Zero-shot
RAG	Yes	No	Yes	GPT series	Zero-shot
BASE	Yes	No	No	GPT series	Zero-shot
TF-IDF + LR	—	—	—	Logistic Regression	Supervised
Legal-BERT	—	—	—	BERT-base	Fine-tuned

GPT series includes GPT-3.5-Turbo, GPT-4o-mini, GPT-4o, and GPT-5.2, evaluated under identical prompt configurations.

### 4.3 Evaluation indicators

The scoring framework proposed in this study evaluates international instrument clauses across three dimensions. The outputs are defined on a five-level ordered discrete scale with a fixed step size of 0.25, with the scoring space specified as (0.0, 0.25, 0.50, 0.75, 1.0). To accurately capture differences in model performance when handling ordinal data, this study employs four evaluation metrics.

#### 4.3.1 Quadratic Weighted Kappa

QWK is the primary metric for evaluating agreement between model predictions and expert annotations, particularly in ordinal rating tasks with graded score levels. The metric assesses prediction quality by measuring the degree to which observed agreement exceeds chance agreement. Its weighting matrix adopts a squared-error scheme, thereby imposing stricter penalties on larger cross-category deviations. For example, misclassifying a score of 1.0 as 0.0 incurs a substantially higher penalty than predicting 0.75, thereby distinguishing minor deviations from severe misclassifications. Higher QWK values indicate better model performance.

#### 4.3.2 Mean Absolute Error

MAE measures the average magnitude of prediction errors by calculating the mean absolute difference between model predictions and human-annotated ground-truth scores. Under the discrete scoring scale used in this study, MAE provides an intuitive measure of the numerical deviation in model predictions, thereby indicating prediction stability. Lower MAE values indicate better model performance.

#### 4.3.3 Spearman's rank correlation (ρ)

ρ is a rank-based nonparametric measure of association that evaluates the strength of monotonic relationships by comparing the rank ordering of predicted scores with that of the ground-truth annotations. In ordinal rating tasks, this metric evaluates whether the model correctly captures the relative ordering of “legalization intensity” across clauses, thereby assessing its ability to discriminate between ordered score levels. Higher values of ρ indicate better model performance.

#### 4.3.4 Exact Agreement Rate

EAR measures the proportion of samples for which the predicted score exactly matches the human-annotated ground-truth label. This metric requires strict matching between predicted values and the five-level discrete labels [(0.0, 0.25, 0.50, 0.75, 1.0)], thereby directly reflecting the model's ability to produce exact label predictions in a five-level ordinal classification task. Higher EAR values indicate better model performance.

Given the high semantic complexity of legal texts, large language models exhibit inherent stochasticity when generating scoring outputs. To ensure robustness and reproducibility of the experimental results, all GPT-series models were evaluated across three independent runs, and the results are reported as mean ± standard deviation (Mean ± SD). In contrast, traditional machine-learning baselines and fine-tuned discriminative models employ deterministic inference mechanisms, enabling stable results from a single run. Accordingly, their standard deviation is recorded as zero to ensure that all methods are compared within a consistent statistical framework.

## 5 Experimental results and discussion

This section systematically reports the experimental evaluation results of the scoring framework proposed in this study and two supervised learning baseline models on the ASEAN test set and the cross-domain test set. Regarding the internal ablation comparison within the framework, under the full framework condition (RAG+CoT), all models except GPT-3.5-Turbo outperform the single-module and baseline conditions across all four evaluation metrics. These results indicate that both the RAG retrieval component and the CoT reasoning module make independent positive contributions to the framework's overall scoring performance and that their synergistic integration can yield significant performance gains. At the cross-method comparison level, GPT-5.2 and GPT-4o operating under the full framework achieve better overall performance on both the ASEAN test set and the cross-domain test set than the supervised TF-IDF+LR model and the fine-tuned Legal-BERT model. This result indicates that, under the experimental settings of this study, the hybrid RAG scoring framework combined with a CoT prompting strategy achieves overall performance superior to that of traditional supervised learning methods under zero-shot conditions. The complete experimental statistical results are summarized in Table 8, and the subsequent sections will provide a systematic, in-depth discussion of each key performance metric.

**Table 8:** Main experimental results of the proposed hybrid RAG framework and two baseline models.

Model	Mode	ASEAN test set				Cross-domain test set			
		QWK	MAE	ρ	EAR	QWK	MAE	ρ	EAR
GPT-5.2	FULL	**0.806 (0.014)**	**0.089 (0.001)**	**0.792 (0.011)**	**0.700 (0.016)**	**0.788 (0.018)**	**0.084 (0.005)**	**0.782 (0.017)**	**0.709 (0.019)**
	COT	0.757 (0.009)	0.115 (0.003)	0.743 (0.008)	0.594 (0.014)	0.754 (0.014)	0.094 (0.003)	0.749 (0.016)	0.676 (0.007)
	RAG	0.719 (0.002)	0.136 (0.002)	0.735 (0.004)	0.558 (0.010)	0.695 (0.006)	0.145 (0.004)	0.731 (0.003)	0.508 (0.015)
	BASE	0.674 (0.008)	0.176 (0.002)	0.712 (0.008)	0.440 (0.003)	0.642 (0.001)	0.166 (0.001)	0.690 (0.004)	0.466 (0.006)
GPT-4o	FULL	**0.788 (0.008)**	**0.098 (0.003)**	**0.770 (0.008)**	**0.684 (0.008)**	**0.762 (0.017)**	**0.096 (0.008)**	**0.742 (0.019)**	**0.671 (0.030)**
	COT	0.731 (0.007)	0.123 (0.003)	0.694 (0.010)	0.591 (0.014)	0.704 (0.019)	0.119 (0.001)	0.675 (0.012)	0.597 (0.028)
	RAG	0.726 (0.003)	0.130 (0.001)	0.729 (0.001)	0.575 (0.002)	0.677 (0.011)	0.137 (0.002)	0.683 (0.013)	0.545 (0.006)
	BASE	0.686 (0.020)	0.156 (0.008)	0.689 (0.025)	0.505 (0.025)	0.655 (0.009)	0.141 (0.002)	0.652 (0.010)	0.536 (0.007)
GPT-4o-mini	FULL	**0.679 (0.017)**	**0.151 (0.007)**	**0.684 (0.017)**	**0.545 (0.017)**	**0.621 (0.002)**	**0.157 (0.004)**	**0.636 (0.010)**	**0.505 (0.019)**
	COT	0.679 (0.019)	0.153 (0.005)	0.672 (0.026)	0.531 (0.010)	0.616 (0.013)	0.162 (0.005)	0.618 (0.016)	0.489 (0.013)
	RAG	0.616 (0.006)	0.159 (0.001)	0.645 (0.004)	0.529 (0.002)	0.533 (0.009)	0.174 (0.005)	0.578 (0.016)	0.473 (0.018)
	BASE	0.526 (0.013)	0.205 (0.004)	0.583 (0.011)	0.401 (0.010)	0.432 (0.012)	0.206 (0.005)	0.487 (0.019)	0.413 (0.019)
GPT-3.5-turbo	FULL	0.512 (0.007)	0.227 (0.001)	0.555 (0.000)	0.354 (0.005)	0.465 (0.015)	0.230 (0.004)	0.523 (0.022)	0.346 (0.011)
	COT	0.530 (0.014)	0.207 (0.006)	0.564 (0.010)	0.389 (0.012)	**0.494 (0.015)**	**0.205 (0.004)**	**0.529 (0.018)**	**0.385 (0.009)**
	RAG	**0.564 (0.002)**	**0.178 (0.000)**	**0.581 (0.014)**	**0.491 (0.004)**	0.445 (0.007)	0.233 (0.002)	0.529 (0.008)	0.358 (0.004)
	BASE	0.398 (0.010)	0.247 (0.002)	0.509 (0.019)	0.336 (0.005)	0.349 (0.008)	0.268 (0.004)	0.468 (0.012)	0.277 (0.007)
TF-IDF + LR	N/A	0.728 (0.000)	0.133 (0.000)	0.734 (0.000)	0.553 (0.000)	0.707 (0.000)	0.138 (0.000)	0.710 (0.000)	0.509 (0.000)
Legal-BERT	N/A	0.667 (0.000)	0.154 (0.000)	0.656 (0.000)	0.518 (0.000)	0.627 (0.000)	0.160 (0.000)	0.629 (0.000)	0.482 (0.000)

Reported values are mean scores across repeated runs, with standard deviations shown in parentheses. Bold values indicate the best performance among the four prompting modes for each model on each test set.

From the experimental results, GPT-5.2 operating under the full framework achieves the best overall performance on both the ASEAN test set and the cross-domain test set, and shows consistent improvements across the four evaluation metrics compared with the Base mode. Taking the ASEAN test set as an example, under the FULL mode, the QWK of GPT-5.2 increases to 0.806 (19.6% higher), ρ increases to 0.792 (11.2% higher), and EAR increases to 0.700 (59.1% higher), while MAE decreases to 0.089 (49.4% lower). Among them, the QWK metric differs from the scoring agreement between two legal experts (0.823) by only 0.017, indicating that the model's scoring results are close to the level of inter-expert agreement. Meanwhile, under the FULL mode, all four evaluation metrics outperform the individual CoT mode and RAG mode, indicating that the retrieval-augmentation mechanism and the chain-of-thought reasoning process play complementary roles in this task. Given that clauses in international legal documents commonly exhibit semantic ambiguity and institutional complexity, GPT-5.2 in Full mode shows strong potential for scoring international legal clauses.

Compared with GPT-5.2, GPT-4o shows slightly lower overall performance on both the ASEAN and cross-domain test sets. This difference may be related to stronger models having more efficient capabilities for integrating retrieved evidence and chain-of-thought reasoning. Nevertheless, GPT-4o operating under the full framework still maintains strong competitiveness across the four evaluation metrics compared with the baseline models. Taking the ASEAN test set as an example, under the FULL mode, GPT-4o achieves QWK, ρ, and EAR scores of 0.788, 0.770, and 0.684, respectively, while MAE decreases to 0.098. Compared with the Base mode, the indicators respectively increase by approximately 14.9%, 11.8%, and 35.4%, while MAE decreases by about 37.2%. And all results significantly outperform the TF-IDF+LR and Legal-BERT baseline models. In addition, GPT-4o and GPT-5.2 exhibit highly consistent result trends across the two test sets, both showing a stable pattern in which FULL outperforms CoT, RAG, and Base. This trend is also verified in the cross-domain test set. This result indicates that although large language models differ in absolute performance, the scoring framework proposed in this study demonstrates strong methodological compatibility and stability across high-capability models.

It is worth noting that the overall performance of GPT-5.2 and GPT-4o declines on the cross-domain test set. Under the FULL mode, the QWK of GPT-5.2 decreases to 0.788 (2.2% lower), and ρ decreases to 0.782 (1.3% lower); the QWK of GPT-4o decreases to 0.762 (3.3% lower), and ρ decreases to 0.742 (3.6% lower). The above phenomenon may be due to a domain shift: the retrieval corpus used in this study primarily consists of ASEAN documents, which are highly consistent with the ASEAN test set in terms of terminology and normative context. In contrast, the documents in the cross-domain test set come from other regional organizations, and the differences in terminology and normative context may cause the embedding model's representations in the latent semantic space to shift, thereby reducing the semantic matching degree of the retrieval results. Nevertheless, GPT-5.2 and GPT-4o still maintain relatively strong performance on the cross-domain test set, and the Full mode continues to outperform single-module settings and supervised learning baselines. At the same time, the MAE of both models and the EAR of GPT-5.2 show slight improvements on the cross-domain test set. The above results indicate that the scoring framework proposed in this study is not limited to ASEAN internal documents and possesses some cross-domain generalization capability. However, the results also indicate that the domain coverage and institutional diversity of the retrieval corpus remain important factors affecting the cross-domain performance of the scoring framework.

In comparison, GPT-4o-mini performs noticeably weaker in the international legal clause scoring task. Compared with GPT-5.2 operating under the full framework, GPT-4o-mini achieves a QWK of 0.679 (15.8% lower), ρ of 0.684 (13.6% lower), EAR of 0.545 (22.1% lower), and MAE of 0.151 (69.7% higher) on the ASEAN test set. This result is slightly higher than the TF-IDF + LR model without deep semantic modeling, but still lower than the domain fine-tuned Legal-BERT model. The main reason for the overall decline in consistency metrics may be that GPT-4o-mini is constrained by a smaller parameter scale and a relatively limited semantic representation, making it more susceptible to superficial linguistic features in complex legal texts and unable to fully leverage similar clauses retrieved by the retrieval module for fine-grained judgment. At the same time, weaker reasoning ability may also limit its consistent application of the scoring criteria under chain-of-thought prompting (Wei et al., 2022). Considering that the inference cost of GPT-4o-mini is significantly lower than that of flagship models, it still has high practical value in large-scale international document scoring tasks where accuracy requirements are moderate or cost sensitivity is high. In addition, the output trend results of GPT-4o-mini on both test sets remain largely consistent with those of GPT-5.2 and GPT-4o, further indicating that the scoring framework proposed in this study is applicable across large language models with different parameter scales.

The convergence observed across the outputs of the three models provides an empirical basis for evaluating the relative contributions of the RAG retrieval module and the CoT reasoning module. Taking GPT-5.2 on the ASEAN test set as an example, the QWK under the CoT mode reaches 0.757, representing an improvement of +0.083 over the baseline (0.674), while the RAG mode achieves a QWK of 0.719, corresponding to an improvement of +0.045. In this setting, CoT's standalone contribution is approximately 1.8 times that of RAG. A similar pattern is observed in GPT-4o (CoT: 0.045 higher vs. RAG: 0.040 higher) and GPT-4o-mini (CoT: 0.153 higher vs. RAG: 0.090 higher). These findings suggest that, within the proposed framework, structured reasoning mechanisms play a more critical role than example-based retrieval in enhancing model scoring performance. Furthermore, in Full mode, GPT-5.2 achieves a QWK of 0.806, representing a 0.132 improvement over the baseline. This exceeds the sum of the individual contributions of CoT (0.083) and RAG (0.045) (0.128 combined), indicating a superadditive interaction effect between the two components. In other words, the two components do not operate independently but instead form a synergistic mechanism targeting complementary failure modes: the RAG module anchors model judgments to semantically relevant, expert-annotated reference clauses, while the CoT module imposes a structured, theory-driven reasoning process that guides the model's decision-making. Their integration ultimately yields synergistic improvements in both accuracy and consistency.

Among all experimental models, GPT-3.5-turbo shows the weakest overall performance in the international legal clause scoring task. Whether on the ASEAN test set or the cross-domain test set, its best performance is significantly lower than that of other large language models and the two supervised learning baseline models. In addition, the output trend of GPT-3.5-turbo also differs significantly from that of other models. Taking the QWK metric as an example, in the ASEAN test set, its performance ranking is RAG > CoT > FULL > Base; while in the cross-domain test set, it appears as CoT > FULL > RAG > Base. This is clearly different from the trend observed in other models, where the FULL mode typically performs best. This phenomenon may be related to GPT-3.5-turbo's limited ability to integrate information when processing longer contexts. Previous research indicates that when the input context becomes long, models tend to focus more on information at the beginning or end of the text, while their ability to utilize information in the middle significantly declines (Hsieh et al., 2024). In this study, the total length of the base instruction and CoT prompting template is usually about 2,500 tokens, and the FULL mode further adds RAG retrieval examples on this basis, increasing the overall input length. Under such circumstances, GPT-3.5-turbo may struggle to reliably locate the key information needed for scoring within a long context, leading to a decline in reasoning quality. In contrast, the RAG or CoT mode containing only a single module has relatively shorter inputs and more focused information, which, to some extent, reduces the information-integration burden caused by long-context processing. This is also an important reason why the RAG and CoT modes of GPT-3.5-turbo achieve the best performance on the ASEAN and cross-domain test sets, respectively.

Through manual review of the model outputs, this study finds that the differences between the model predictions and expert annotations can mainly be attributed to two aspects:

The first important reason is that the scoring criteria in this study follow a relatively linear decision process, which has limitations when dealing with boundary clauses that contain semantic ambiguity or multiple normative functions. Taking Clause 38 in the ASEAN test set as an example: “The participating countries shall extend to an existing AIJV product the same tariff preferences… within 90 days from the date the AEM approves the inclusion of that product in the final list.” According to the decision procedure for the obligation dimension, the key branching point occurs at Step 3, which asks whether “exceptions to the obligation exist or whether the obligated actor is not a contracting party.” This clause makes “AEM approval” a prerequisite for the obligation's activation. Such wording may be interpreted as a procedural institutional prerequisite for implementing the obligation, without weakening the obligation itself. It may also be interpreted as a substantive limitation on the scope of the obligation. Annotators may be more inclined toward the first interpretation, viewing the clause as a clear obligation accompanied by a procedural condition and thus assigning a score of 0.75. In contrast, the model may interpret “AEM approval” as an implicit exception condition, meaning that the obligation only takes effect upon prior approval from an external institution, and therefore assign a score of 0.5. The fundamental reason for this divergence is that the scoring criteria lack a clear definition of whether procedural preconditions constitute exceptions to obligations. Similar interpretive differences also exist in the precision and delegation dimensions. This reflects the structural challenges faced by rule-based scoring frameworks when dealing with real legal texts and also represents a potential source of error that rule-based automated annotation methods must address in complex legal contexts.

The second important reason is that the scoring framework proposed in this study uses a single clause as the basic input unit, which leads the model to lack relevant contextual information and cross-clause institutional background during clause-by-clause evaluation. This limitation causes the model to produce interpretive deviations when handling clauses whose normative nature requires contextual information for accurate judgment, due to incomplete input information, thereby affecting the scoring accuracy. Taking Clause 143 in the ASEAN test set as an example: “The AHTN shall be an 8-digit nomenclature, consisting of the latest version of the Harmonized Commodity Description and Coding System…” When reading the full document, annotators can identify from the title and preface that AHTN (ASEAN Harmonized Tariff Nomenclature) is a technical tariff classification standard. Considering that the clause appears in a definitions section, annotators can correctly recognize “shall be an 8-digit nomenclature” as a descriptive definition of a technical standard rather than a normative requirement for member state behavior, and therefore assign a score of 0.0. In contrast, when processing sentences individually, the model cannot access the document title or structural chapter information and can only observe that “AHTN” and “shall” appear together in the sentence, leading it to interpret the expression as a legally binding normative statement and assigning a score of 0.75. Similar deviations also occur in the delegation and precision dimensions. It can therefore be seen that when the normative nature of a clause requires the accurate identification of document structure, terminology definitions, or cross-clause institutional background, a clause-level input scoring framework may encounter information gaps, leading to deviations in scoring results.

Nevertheless, the scoring framework proposed in this study still offers several methodological advantages for international legal clause evaluation. Compared with traditional supervised learning models, this framework does not rely on any labeled data and can generate step-by-step reasoning through CoT prompting to complete the scoring process. When a small amount of labeled data is available, the retrieval corpus can also be expanded through the RAG module, thereby forming a flexible extension mechanism between zero-shot and few-shot settings. In contrast, both TF-IDF+LR and Legal-BERT rely on supervised training to achieve usable performance, and their applicability is relatively limited in scenarios where labeled data are scarce or cross-domain transfer is required. In addition, the CoT prompting template embedded in this framework can generate traceable reasoning chains, which is of great significance for manual verification and quality control of annotation. In comparison, TF-IDF+LR mainly relies on surface-level lexical features, whereas Legal-BERT, although showing strong performance in the domain, has a decision-making process that is difficult to interpret and examine. In legal research contexts, interpretability itself has important methodological value, especially in scenarios where large-scale annotation tasks require human–machine collaboration. From the perspective of legalization research, the scoring framework proposed in this study offers a potential methodological pathway for conducting large-scale empirical analysis of the degree of legalization in international legal documents and helps improve measurement comparability and reproducibility of results across studies.

## 6 Application: measuring the degree of legalization of ASEAN instruments

The hybrid scoring framework proposed in this study is essentially a quantitative tool suitable for macro-institutional analysis and can measure the degree of legalization of international instruments. Simply put, after receiving a substantive clause text as input, the system generates scores covering the three dimensions of obligation, precision, and delegation, along with reasoning explanations. After aggregating the scoring results for all clauses within a given international instrument, a composite legalization index can be calculated using methods such as linear weighted aggregation, principal component analysis, or latent variable modeling. This index provides quantitative evidence for examining the level of institutionalization of inter-state cooperation mechanisms, identifying factors influencing institutional arrangement choices, and understanding the historical evolution of institutional design.

**Figure 3:**
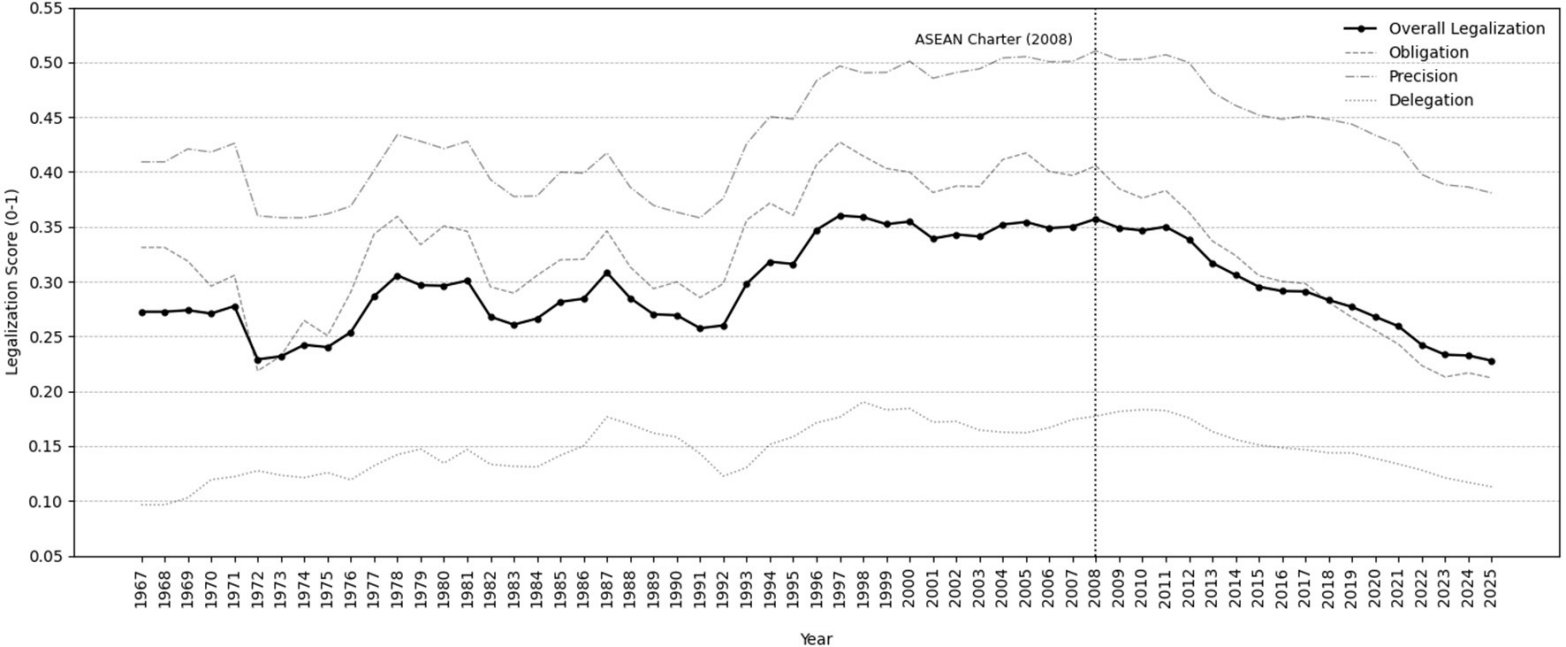
Trends in the legalization index of ASEAN instruments and the three-dimensional scores, 1967–2025.

To more clearly demonstrate the framework's application, this study applies it to measuring the degree of legalization of ASEAN instruments. To this end, this study constructs a dataset covering 1,273 ASEAN instruments and 30,096 clauses, broadly covering major official documents issued since the establishment of ASEAN. Based on the hybrid scoring framework, this study conducts a clause-by-clause quantitative evaluation of 30,096 clauses and assigns scores to each clause across the three dimensions of obligation, precision, and delegation. On this basis, this study further introduces the Graded Response Model (GRM) for each dimension, treating each clause as an ordered multi-category measurement item and estimating the latent trait score for each dimension at the instrument level. The GRM is specifically designed for ordered multi-category response data; it can model differences in item discrimination and cumulative threshold structures, and helps improve the precision and robustness of latent trait estimation (Samejima, 1969). Subsequently, following the weights proposed by Abbott et al. (2000) for the three dimensions of legalization, this study calculates the composite legalization index for each instrument using a weighted average. Finally, this study uses the annual average scores for the three dimensions and the composite legalization index as time-series indicators, and applies a 5-year moving average to plot line charts that present the changing trend in the legalization of ASEAN instruments. The specific results are shown in Figure 3.

Overall, the degree of legalization of ASEAN instruments is generally low. Across the three dimensions, precision scores have consistently ranked highest over the years, followed by obligation, while delegation has remained at the lowest level. This structural feature corroborates the “ASEAN Way” identified in previous studies, namely non-interference in internal affairs, respect for member state sovereignty, and the principle of consensus (Natalegawa, 2018; Jones and Smith, 2007). From the perspective of institutional design logic, ASEAN's normative orientation, emphasizing political flexibility and member-state autonomy, has limited the formation of institutional arrangements with stronger legal binding force, keeping the overall level of legalization relatively low (Collins, 2019). In the long-term trend, from 1967 to 2008, the degree of legalization of ASEAN instruments generally showed a continuous upward trend; However, after the promulgation of the 2008 ASEAN Charter, its upward trajectory showed a clear turning point. These findings are also consistent with previous studies, which argue that ASEAN's institutionalization process is not linear but characterized by phased fluctuations. Overall, the degree of legalization of its instruments has undergone a dynamic process, from an initially low level to a gradual increase, followed by subsequent adjustment. The hybrid scoring framework proposed in this study demonstrates methodological utility in analyzing ASEAN institutional practice and provides an operational tool for research on international instrument design and regional governance.

To facilitate its application in empirical legal research, the hybrid scoring framework proposed in this study can be deployed as a web-based decision support system. Within this deployment environment, users can batch-upload document data via an online interface, through which the system automatically generates scores across the three dimensions of “legalization,” accompanied by corresponding reasoning explanations. The interface may further support visualization of score distributions, traceable reasoning chains, and structured export of results, thereby lowering the barriers for researchers in international law and international relations to conduct large-scale empirical analyses. It should be noted that when applied to legal reasoning tasks, LLMs may introduce trust-related risks due to instability in reasoning, context sensitivity, and potential training biases. The WASABI model proposed by Singh and Singh emphasizes that such risks must be systematically addressed through responsible design practices, including accountability, transparency, and robustness (Singh and Singh, 2023). Accordingly, the deployment and application of this framework should adhere to the seven key requirements outlined in the Assessment List for Trustworthy Artificial Intelligence (ALTAI) (Radclyffe et al., 2023). In line with these principles, the scoring framework incorporates multiple safeguard mechanisms within its methodological design:

First, this framework introduces a RAG mechanism to reduce bias risks posed by general large language models due to differences in pre-training corpus distributions. This mechanism can construct an external reference structure for the model's reasoning process by integrating semantically similar expert-annotated examples. When extending to other regional treaty systems, the reasoning shift caused by cross-regional contextual differences can be mitigated by constructing and expanding expert-annotated semantic databases in the corresponding domain. It should be clarified that the international instruments used in this study all originate from publicly available databases and do not contain personal or sensitive information; therefore, there are no privacy risks to personal data.

Second, the CoT prompt template adopted in this framework embeds the legalization conceptual framework proposed by Abbott et al. at the design level, transforming its three-dimensional structure into an executable reasoning path. This prompt template can guide the model to sequentially evaluate each dimension according to predefined coding standards, generating corresponding scoring results and reasoning explanations. Through the above stepwise decision mechanism, this framework achieves a consistent mapping of “normative framework–reasoning path–scoring output”, thereby reducing potential leap-of-reasoning and superficial semantic-matching problems in the model and lowering the risk of hallucinations.

In addition, during the practical deployment stage, this framework will adopt strict version control and parameter locking mechanisms to reduce the risk of output drift caused by model iteration, prompt fine-tuning, or changes to LLM inference parameters. At the same time, the framework will introduce logging and result caching mechanisms, generating timestamp identifiers, model signature information, and parameter snapshots for each inference call, and storing structured input, output, and reasoning trace data. This mechanism enhances the reproducibility and traceability of scoring results at the technical level and provides the necessary technical foundation for cross-version comparison and retrospective analysis of model outputs.

## 7 Limitations and future work

In addition to the limitations discussed in the discussion section, the expert-designed rule system used in this study also has subjective constraints. Based on the legalization theoretical framework proposed by Abbott et al., this study constructed a five-level coding system and a stepwise decision-making process, and used it as a CoT prompting template to improve the transparency and auditability of the model scoring process. However, this coding method, both in its design and application, still relies on experts' interpretation of rule meanings and experiential judgment of borderline clauses, thus making it difficult to completely avoid the influence of subjective factors. This subjectivity may not only affect the consistency between model scoring and expert annotations but also introduce systematic bias across annotators. It should be emphasized that this limitation is not unique to this study, but is a common challenge faced by all content analysis methods based on expert rule systems constructed from theoretical frameworks, rooted in the semantic ambiguity and interpretive space of legal texts themselves. Although such biases are difficult to eliminate, they can be mitigated through methodological design. To this end, future research should, as much as possible during the rule system design stage, ensure the objectivity and reproducibility of standards, and introduce multiple annotators to conduct parallel coding of the same samples, resolving disagreements through multi-round arbitration mechanisms, thereby reducing the influence of subjective factors on scoring results.

The second limitation of this study concerns the constraints inherent in the clause-level input architecture. Clause-level analysis of legal documents is a common approach in empirical international law research, as it enables a more fine-grained characterization of heterogeneity within institutional design and mitigates the dilution of key normative content inherent in whole-text representations. However, this design may lead to the omission of inter-clause dependencies and broader institutional context when the model evaluates clauses individually. In contrast, human experts typically incorporate such information into their assessments. As demonstrated in the error analysis, when the normative nature of a clause can be accurately determined only by reference to surrounding provisions, definitional clauses, or the document's overall institutional design, this limitation may result in systematic scoring bias. Although CoT prompting and RAG can partially alleviate this limitation, neither can fully substitute for the document-level institutional reasoning performed by human experts. Accordingly, future research may explore hierarchical input architectures that integrate document-level structural metadata alongside clause text, offering a feasible intermediate solution for incorporating institutional context without compromising scalability.

The third limitation of this study concerns the corpus's limited scope. The vector database underlying the RAG module in the current scoring framework is constructed exclusively from ASEAN legal documents and does not yet incorporate materials from the European Union, the African Union, or other international organizations. Although experimental results on a cross-domain test set suggest that the framework demonstrates some out-of-domain applicability, the limited size and scope of this dataset are insufficient to fully capture the normative logics and linguistic patterns of other institutional systems. Substantial differences often exist across international organizations in institutional design, legal obligation structures, and treaty drafting practices; consequently, semantic representations and scoring references derived from ASEAN documents may not always generalize effectively to texts in other legal and institutional contexts. In particular, when the scoring framework is applied to large-scale corpora spanning multiple institutional systems, such discrepancies may introduce systematic biases, thereby affecting the interpretation and analytical validity of cross-organizational comparisons. To address this limitation, future research should expand the corpus used to construct the vector database by incorporating legal documents from a broader range of issue areas and institutional systems. This would enhance the model's adaptability to diverse legal styles, institutional structures, and normative logics and improve the reliability of the framework in large-scale cross-domain analytical settings. Additionally, hybrid retrieval strategies that combine lexical and dense approaches, along with modular classification-based routing mechanisms, can be incorporated to enhance the framework's cross-context generalizability and improve retrieval robustness.

Despite the aforementioned limitations, the scoring framework proposed in this study can still provide effective technical support for large-scale empirical analyses of legalization in international legal instruments. Under the optimal configuration, GPT-5.2 and GPT-4o both achieve high levels of consistency, indicating that the scoring framework demonstrates strong stability and reliability in evaluating clauses in international legal instruments. Future research may employ this framework to automatically score legal documents signed over time by ASEAN or other international organizations, thereby tracing the longitudinal evolution of their institutionalization processes. Building on this, researchers may further integrate regression analysis or causal inference methods to examine how political, economic, and institutional factors shape the institutionalization process of such organizations, thereby advancing legalization theory from a predominantly descriptive stage toward more explanatory causal analysis. In addition, researchers may align legalization scores for international legal instruments with existing treaty compliance databases to systematically examine the substantive impact of legalization levels on treaty compliance rates, thereby providing large-scale empirical evidence for the theory's core propositions. In summary, the scoring framework proposed in this study provides a scalable methodological tool for large-scale, cross-organizational comparative research on legalization in international legal instruments, facilitating the transition of legalization theory from traditional qualitative analysis toward systematic quantitative inquiry.

## 8 Conclusion

This study proposes a scoring framework that integrates CoT prompting with a hybrid RAG pipeline to quantitatively evaluate the degree of legalization in international legal instruments across three dimensions: obligation, precision, and delegation. In terms of interpretability, the embedded CoT prompting templates generate traceable, step-by-step reasoning chains for each scoring decision, ensuring a high degree of transparency and auditability in legal research contexts involving human–machine collaboration. In terms of scalability, the RAG module enables flexible domain adaptation and performance improvement by expanding the retrieval corpus within the vector database, without requiring parameter updates, thereby supporting a gradual transition from zero-shot to few-shot settings. These features position the framework as a scalable and transparent methodological tool for large-scale empirical analyses of legalization in international instruments, with strong potential for application across diverse treaty systems and regional organizations.

Experimental results on both the ASEAN and cross-domain test sets demonstrate that GPT-5.2 and GPT-4o operating under the full framework significantly outperform the base prompting strategy, the trained TF-IDF+LR model, and the fine-tuned Legal-BERT across all four evaluation metrics, with GPT-5.2 achieving the best overall performance. On the ASEAN test set, GPT-5.2 achieves a QWK of 0.806 under the optimal configuration, only 0.017 below the inter-expert agreement score of 0.823, indicating that its scoring consistency approaches human expert levels. Ablation experiments further confirm that the RAG retrieval module and the CoT reasoning module each contribute independently and complementarily to overall performance, with their integration yielding substantial performance gains. Taken together, these findings validate the effectiveness of the proposed framework and demonstrate that integrating structured theoretical guidance with dynamic knowledge retrieval can significantly enhance the stability of reasoning and consistency of scoring for large language models in complex legal text evaluation tasks.

To demonstrate the empirical utility of the proposed scoring framework, this study further applies it to a quantitative analysis of 30,096 clauses drawn from 1,273 ASEAN legal documents. The results show that, among the three dimensions of legalization, precision consistently records the highest scores over time, followed by obligation, while delegation remains at the lowest level. The long-term trend exhibits an overall increase from 1967 to 2008, followed by a clear turning point after the promulgation of the ASEAN Charter in 2008. These findings are highly consistent with existing literature describing the institutional characteristics of the “ASEAN Way” and the non-linear nature of its institutionalization trajectory. They provide quantitative empirical support for the proposition that ASEAN institutional design prioritizes political flexibility and member state autonomy. They also demonstrate that the proposed framework has substantial methodological utility in large-scale analyses of regional institutional practices.

This study also acknowledges several limitations of the proposed framework. The current vector database covers only ASEAN documents, and the clause-level input design prevents the model from accessing cross-clause institutional context. At the same time, the scoring rule system relies on expert judgment, making it difficult to eliminate subjective influences. These limitations may introduce systematic scoring biases under certain conditions. Future research may address these limitations along two main directions: first, at the level of rule system design, incorporating multi-annotator coding and adjudication mechanisms can enhance objectivity and reproducibility, while exploring input architectures that integrate document-level contextual information. Second, at the level of corpus coverage, expanding the retrieval database to include international legal documents from other regional and global organizations such as the EU, AU, and the UN can enhance the framework's cross-domain applicability.

## Data Availability

The datasets presented in this study can be found in online repositories. The names of the repository/repositories and accession number(s) can be found below: https://github.com/Rwabhineda/A-Hybrid-RAG-Scoring-Framework-Based-on-Chain-of-Thought-Prompting.

## Footnotes

1 Center for International Law. Database-CIL. National University of Singapore. Website. Accessed August 18, 2025. Available from: https://cil.nus.edu.sg/database-cil/.

2 https://github.com/Rwabhineda/A-Hybrid-RAG-Scoring-Framework-Based-on-Chain-of-Thought-Prompting.

3 The corresponding API model identifiers used in this study are as follows: gpt-3.5-turbo-0125 (for GPT-3.5-Turbo), gpt-4o-mini-2024-07-18 (for GPT-4o-mini), gpt-4o-2024-08-06 (for GPT-4o), and gpt-5.2-2025-12-11 (for GPT-5.2). All models were accessed via the OpenAI API.

## Supplementary material

Appendix Figure A1 Structure of the base prompt module.

Appendix Figure A2 Structure of the RAG prompt module.

Appendix Figure A3 Structure of the CoT prompt module.
