# Correction: The legalization of international instruments: a hybrid RAG scoring framework based on chain-of-thought prompting

**DOI:** 10.3389/frai.2026.1932998

**Published:** 2026-07-22

**Authors:** Yan Chen, Zihua Zeng, Muhamad Sayuti Hassan

**Affiliations:** 1 Faculty of Law, Universiti Kebangsaan Malaysia, Bangi, Malaysia; 2 School of Computer Science, The University of Sydney, Sydney, NSW, Australia

**Keywords:** chain-of-thought prompting, international instruments, large language models, legalization, retrieval-augmented generation

Authors Yan Chen and Zihua Zeng were erroneously omitted as equal contributing authors.

The Equal Contribution Statement for Yan Chen and Zihua Zeng was omitted from the published article. The correct statement is: “Yan Chen and Zihua Zeng contributed equally to this work and share first authorship.”

The original version of this article has been updated.

